# Distinct axial and lateral interactions within homologous filaments dictate the signaling specificity and order of the AIM2-ASC inflammasome

**DOI:** 10.1038/s41467-021-23045-8

**Published:** 2021-05-12

**Authors:** Mariusz Matyszewski, Weili Zheng, Jacob Lueck, Zachary Mazanek, Naveen Mohideen, Albert Y. Lau, Edward H. Egelman, Jungsan Sohn

**Affiliations:** 1 Department of Biophysics and Biophysical Chemistry, Johns Hopkins University School of Medicine, Baltimore, MD USA; 2 Department of Biochemistry and Molecular Genetics, University of Virginia School of Medicine, Charlottesville, VA USA; 3 Present Address: Department of Cellular and Molecular Medicine, University of California San Diego, La Jolla, CA USA

**Keywords:** Cryoelectron microscopy, Computational models

## Abstract

Inflammasomes are filamentous signaling platforms integral to innate immunity. Currently, little is known about how these structurally similar filaments recognize and distinguish one another. A cryo-EM structure of the AIM2^PYD^ filament reveals that the architecture of the upstream filament is essentially identical to that of the adaptor ASC^PYD^ filament. In silico simulations using Rosetta and molecular dynamics followed by biochemical and cellular experiments consistently demonstrate that individual filaments assemble bidirectionally. By contrast, the recognition between AIM2 and ASC requires at least one to be oligomeric and occurs in a head-to-tail manner. Using in silico mutagenesis as a guide, we also identify specific axial and lateral interfaces that dictate the recognition and distinction between AIM2 and ASC filaments. Together, the results here provide a robust framework for delineating the signaling specificity and order of inflammasomes.

## Abstract (web summary)

AIM2-ASC inflammasomes are filamentous signalling platforms that play a central role in host innate defence. Here, the authors present the filament cryo-EM structure of the inflammasome receptor AIM2, which is very similar to the adaptor ASC filament structure. By employing Rosetta and Molecular Dynamics simulations the authors provide further insights into the directionality and recognition mechanisms of the individual AIM2 and ASC filaments, which is further validated with biochemical and cellular experiments.

## Introduction

Inflammasomes are filamentous signaling platforms and play key roles in the metazoan innate immune system^1^. These supra-structures assemble upon detecting molecular signatures arising from various intracellular catastrophes, such as genomic instability, dysfunctional organelles, and pathogen invasion^1^. Mammals have at least 15 different receptors that lead to the assembly of inflammasomes whose ultimate goal is to induce the polymerization of procaspase-1, activating the zymogen protease by proximity-induced autoproteolysis^1^. Caspase-1 then executes two key innate immune responses: the cleavage/maturation of pro-inflammatory cytokines, such as interleukin-1β and -18, and the initiation of pyroptosis^1^. Inflammasomes play essential roles in host defense against pathogen invasion (e.g., coronaviruses, herpesviridae, and *Listeria monocytogene*)^1–6^. In addition, malfunctioning inflammasomes promote acute and chronic autoinflammatory diseases (e.g., severe COVID-19, rheumatoid arthritis, and systemic lupus erythematosus (SLE))^7–9^, metabolic disorders (type 2 diabetes)^10,11^, and even tumorigenesis (colon cancer, lung cancer, and oral cancer)^12,13^.

Inflammasome receptors contain multiple functional domains for autoinhibition, signal recognition, and oligomerization^1,14^. Importantly, the N-terminal pyrin domain (PYD) acts as the primary signal transduction module in the vast majority of inflammasomes^1,14,15^. PYDs are six-helix bundles that belong to the death-domain (DD) superfamily and can assemble into helical filaments. For instance, incoming signals such as viral nucleic acids induce the assembly of a receptor PYD filament^14,16–18^. The upstream PYD filaments then nucleate the filamentation of the PYD of central adaptor ASC (ASC^PYD^)^14,18–20^, leading to the oligomerization of the CARD of ASC (ASC^CARD^) to recruit and trigger the polymerization (activation) of procaspase-1 (ASC: apoptosis-associated speck-forming protein containing caspase-recruiting domain (CARD); CARDs are also six-helix bundles that belong to the DD family)^14,18,21,22^.

Although the structural mechanisms by which inflammasomes assemble are increasingly better understood^14,17–25^, little is known about the mechanisms that direct the signaling order (sequence) and specificity. For instance, all published cryo-electron microscopy (cryo-EM) structures of PYD filaments show essentially the same helical architectures (six subunits per helical turn)^18,20^; all CARD filaments also show the same helical architectures (four subunits per helical turn)^14,21,23^. These observations then led to a well-accepted model, in which the architectural complementarity between upstream and downstream filaments underpins the recognition^14,17,18,20,21,26^. However, it raises a considerably more complex problem as to how these similar helical filaments built from homologous protomers distinguish and recognize one another within respective subfamilies. Here, we address this fundamental mechanistic issue in the cytosolic double-stranded (ds)DNA-sensing AIM2-ASC inflammasome^27^. AIM2 (absent in melanoma 2) is a bipartite protein composed of the N-terminal PYD followed by the dsDNA-binding HIN domain (hematopoietic interferon-inducible nuclear antigen). Upon binding cytosolic dsDNA via its HIN domain, AIM2^PYD^ assembles into filaments, inducing the polymerization of ASC^12,16,17,19^. AIM2 is essential for the host defense against numerous pathogenic viruses and bacteria^6,12,16,27–30^. AIM2 also plays vital roles in neuronal development by regulating timely cell death^31^. However, dysregulated AIM2 leads to various maladies, such as SLE, chronic kidney diseases, and lung cancer^12,27,32–34^.

We present a cryo-EM structure of the AIM2^PYD^ filament at 3.2 Å resolution, which reveals that its architecture is indeed identical to that of the ASC^PYD^ filament. Using our structure, we then investigate how AIM2^PYD^ and ASC^PYD^ filaments recognize, and distinguish each other by Rosetta and molecular dynamics (MD) simulations. Our in silico analyses consistently suggest that the energy landscapes that underpin the assembly of individual filaments do not impose directionality. By contrast, the energy landscape that governs the recognition between AIM2^PYD^ and ASC^PYD^ is polarized in a head-to-tail manner. Multiple biochemical experiments corroborate that individual filaments assemble bidirectionally. Moreover, AIM2^PYD^ and ASC^PYD^ filaments do not co-assemble, and the signal transduction from AIM2 to ASC occurs unidirectionally only when at least one is oligomeric. Using Rosetta-based in silico mutagenesis as a guide, our biochemical and cellular experiments consistently show that lateral interfaces of AIM2^PYD^ drive its bidirectional assembly. We also identify specific axial interfaces that mediate the recognition between AIM2^PYD^ and ASC^PYD^. Together, we demonstrate that distinct interfaces within homologous filaments direct signaling order and specificity of inflammasomes. We also set forth a broadly applicable multidisciplinary platform for delineating the signal transduction order, specificity, and directionality of filamentous assemblies.

## Results

### The cryo-EM structure of AIM2^PYD^

Using EM of negatively stained samples (nsEM), we previously found that the helical symmetry of the AIM2^PYD^ filament is consistent with that of the ASC^PYD^ filament^17^, and thus proposed that architectural complementarity is important for their recognition. However, the published high-resolution cryo-EM structure of the AIM2^PYD^ filament displays an altered helical architecture because the N-terminal green fluorescence protein (GFP)-tag interferes with assembly^35^. Thus, we first determined the cryo-EM structure of the AIM2^PYD^ filament using an untagged recombinant protein.

**Fig. 1:**
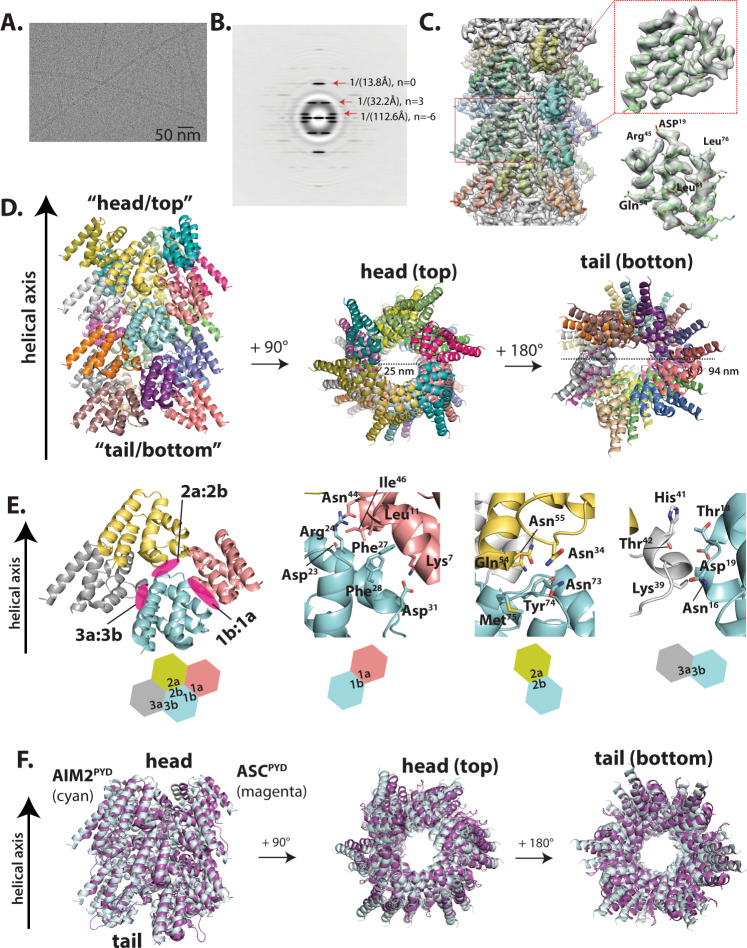
AIM2^PYD^ assembles into an architecturally congruent filament as the ASC^PYD^ filament. **A** A sample cryo-electron micrograph of the AIM2^PYD^ filament (total 976 micrographs were taken). **B** An average power spectrum of the AIM2^PYD^ filament from 512 px-long nonoverlapping segments. **C** The AIM2^PYD^ filament model built into the EM map. A subunit with visible side chains is shown. **D** The model of AIM2^PYD^ filament. Each subunit is colored differently. **E** A cartoon representation of three unique filament interface types. Side chains at each filament interface are shown as a stick configuration. **F** Overlays between AIM2^PYD^ and ASC^PYD^ (PDB ID: 3J63) filaments.

Cryo-EM images showed that AIM2^PYD^ filaments are straight helical rods (Fig. 1A). The average power spectrum of 512-pixel-long nonoverlapping filament segments showed that the AIM2^PYD^ filament displays a six-start, C3 helical symmetry of 54.4° rotation (~6 subunits per helical turn) and an axial rise of 14 Å (Fig. 1B). These parameters are remarkably similar to those of the ASC^PYD^ filament^18^, further solidifying the concept that the upstream receptors provide structural templates for downstream assemblies in inflammasomes^14,17,26^. We fit the crystal structure of AIM2^PYD^ into the EM map for initial modeling^36^, and the refined high-resolution map allowed us to model in most bulky and aliphatic side chains (Fig. 1C). The resolution of the final model was 3.2 Å according to the gold standard method (Supplementary Fig. 1A). The diameter of the outer rim is ~94 Å and that of the inner cavity is ~25 Å (Fig. 1D). The structure of individual AIM2^PYD^ protomers is identical to the crystal structure of AIM2^PYD^ monomer (Supplementary Fig. 1B), thus indicating that, unlike the PYD of NLRP6 (ref. ^20^), an AIM2^PYD^ monomer does not undergo any conformational changes during activation. As seen from the ASC^PYD^ filament, each AIM2^PYD^ subunit contributes three unique protein–protein interaction interfaces (Fig. 1E). The type 1a:1b interface is largely composed of side-chain interactions, while the type 2a:2b and type 3a:3b interfaces involved both side-chain and backbone interactions (Fig. 1E). We also noted several side chains previously implicated in filament assembly throughout different interfaces (e.g., L11, D19, F27, and I46; Fig. 1E)^17^. Aligning the new AIM2^PYD^ filament to the GFP-AIM2^PYD^ filament demonstrates that although the lateral interactions are largely conserved, the axial positions are significantly different due to the altered helical symmetry (five subunits per turn in the GFP-tagged filament vs. six subunits per turn in the untagged filament; Supplementary Fig. 1C). On the other hand, aligning the cryo-EM structures of AIM2^PYD^ and ASC^PYD^ filaments demonstrates their congruent architectures (Fig. 1F). The subtle difference in subunit positions between AIM2^PYD^ and ASC^PYD^ filaments along the helical axis could reflect the unique side-chain interactions that mediate their respective filament assembly or the inherent flexibility of biomolecular structures (Fig. 1F). Nevertheless, the near perfect architectural complementarity between AIM2^PYD^ and ASC^PYD^ filaments supports the idea that upstream filaments provide structural templates for the assembly of downstream filaments^14,17,26,37^.

### Deciphering the specificity and directionality of the AIM2-ASC inflammasome using Rosetta and MD

**Fig. 2:**
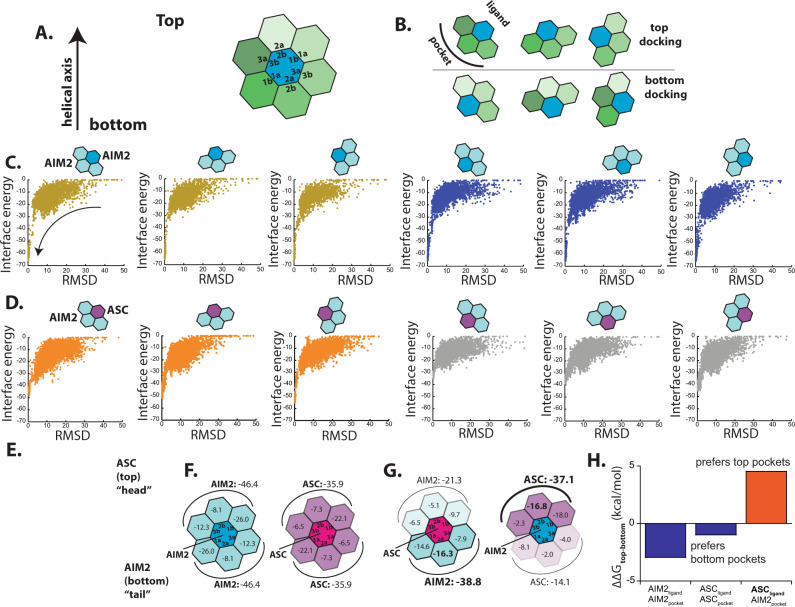
In silico studies suggest that homotypic filaments assemble bidirectionally and the recognition between AIM2 and ASC occurs unidirectionally. **A** Cartoon representations of honeycombs. Each interface type is labeled in the hexagons which represent PYD monomers. **B** Rosetta docking strategy. Top docking indicates that a ligand monomer docks onto the top surface of the pocket, while bottom docking is the opposite. **C** Plots of Rosetta interface energy scores vs. RMSD for top and bottom docking results for AIM2^PYD^ assembly. The **B** represents simulations conducted in **C**. **D** Plots of Rosetta interface energy scores vs. RMSD for docking an ASC^PYD^ monomer on the top or bottom of AIM2^PYD^ pockets. **E** A model of the AIM2^PYD^ filament recognition of the ASC^PYD^ filament. **F**, **G** Rosetta interface energy scores at individual filament interfaces for homotypic and heterotypic assemblies. AIM2^PYD^-AIM2^PYD^, ASC^PYD^-ASC^PYD^, ASC^PYD^-AIM2^PYD^, and AIM2^PYD^-ASC^PYD^. Each hexagon represents AIM2^PYD^ or ASC^PYD^ monomer. **H** A plot for the difference in free energy (∆∆G) for dissociating ligand PYDs from the top or bottom pockets.

AIM2^PYD^ and ASC^PYD^ monomers are homologous and structurally highly conserved (root-mean-squared-deviation, RMSD 0.5 Å), and our new cryo-EM structure shows that they indeed assemble into essentially identical filaments (Fig. 1F). These observations raise significantly more complex questions as to whether and how these supra-structures distinguish and recognize each other. Importantly, such questions are germane to all filamentous signaling platforms employing PYDs or CARDs^21,22,26,37–39^. Thus, to establish a broadly relevant method for tackling these questions, we employed a computational approach using Rosetta. First, we tested whether RosettaDock^40^ could recapitulate the cryo-EM structures by docking an AIM2^PYD^ monomer into our AIM2^PYD^ filament structure (also an ASC^PYD^ monomer to the ASC^PYD^ filament (PDB ID: 3J63)^18^). For instance, each PYD protomer provides three unique interfaces in AIM2^PYD^ and ASC^PYD^ filaments (i.e., six distinct surfaces; Figs. 1E and 2A). To facilitate docking experiments, we generated a honeycomb-like side view of AIM2^PYD^ and ASC^PYD^ filaments, in which the center protomer makes all six possible contacts (Fig. 2A). We then divided the honeycomb into six unique subsections consisting of one ligand docked into a pocket created by three adjacent subunits (Fig. 2B). Using the local docking method in Rosetta^40^, we performed 5000 independent docking simulations between a ligand–pocket pair from each subsection, then compared the interface energy and RMSD from the cryo-EM structures.

For each filament, both parameters decreased concurrently, while displaying uniform energy scores from all subsections (Fig. 2C (arrow) and Supplementary Fig. 2A), indicating that RosettaDock can recapitulate the cryo-EM structures. The more favorable energy scores from the AIM2^PYD^ filament suggest that it is is more stable than the ASC^PYD^ filament (typically −70 s for AIM2^PYD^ complexes vs. −50 s for ASC^PYD^ complexes). Importantly, the uniform energy scores throughout the top and bottom subsections (Fig. 2C and Supplementary Fig. 2A) suggest that individual filaments would assemble bidirectionally. Next, we docked an ASC^PYD^ monomer (ligand) onto all six pockets of the AIM2^PYD^ filament and vice versa (Fig. 2D and Supplementary Fig. 2B). We first noted that the interface energies are not as favorable as the AIM2^PYD^•AIM2^PYD^ complexes (−60 or worse for ASC^PYD^•AIM2^PYD^complxes; Fig. 2D and Supplementary Fig. 2B). Moreover, docking ASC^PYD^ on the top pockets of the AIM2^PYD^ filament was significantly more favorable than docking at the bottom. (Fig. 2D orange vs. gray). Docking AIM2^PYD^ on the ASC^PYD^ filament also showed that AIM2^PYD^ prefers the bottom half of the ASC^PYD^ filament with the energy scores as favorable as the homotypic ASC^PYD^ assembly (Supplementary Fig. 2A, B (2B orange vs. gray)). These results suggest that individual filaments assemble bidirectionally, while the recognition between AIM2^PYD^ and ASC^PYD^ occurs unidirectionally, where the top of the AIM2^PYD^ filament recognizes the bottom of the ASC^PYD^ filament (Fig. 2E).

Next, we used Rosetta InterfaceAnalyzer^41^ to evaluate the interaction energies between homotypic and heterotypic interactions at the individual interfaces of the honeycomb (Fig. 2F). The AIM2^PYD^ complex also showed the most favorable overall interface energy scores (Fig. 2G). Moreover, for the respective homotypic assembly of AIM2^PYD^ and ASC^PYD^, the type 1 interface contributed most significantly with the top and bottom halves displaying symmetric energy scores (Fig. 2F). On the other hand, the interface energy scores were consistently worse, when ASC^PYD^ was placed at the center of the AIM2^PYD^ honeycomb, except for the one between the type 2a of ASC^PYD^ and 2b of AIM2^PYD^ (Fig. 2F vs. 2G). The overall energy scores between the top and bottom halves were again asymmetric, preferring a head-to-tail-like direction, in which the top of AIM2^PYD^s favoring the bottom of ASC^PYD^ and vice versa (Fig. 2E, G; the small difference in energy scores between Fig. 2F, G likely stemmed from the subtle architectural differences in two filaments).

To test whether the simulation results are not biased by a particular algorithm, we then used MD to calculate the free energy required to dissociate a ligand PYD from each pocket described in Fig. 2B (i.e., stability; Supplementary Fig. 2C, D). Consistent with the results from RosettaDock, MD simulations suggested the AIM2^PYD^ filament complex to be most stable, followed by ASC^PYD^•AIM2^PYD^ then ASC^PYD^ complexes. (Supplementary Fig. 3B; see also Supplementary Fig. 3C for images showing the dissociation of each monomer before and after the simulation). We compared the sum of energies required to dissociate a ligand PYD from the top vs. bottom halves (Fig. 2H; Supplementary Fig. 2C). Individually, AIM2^PYD^ and ASC^PYD^ complexes showed mostly uniform energy landscapes from either filament pole, with both filaments showing more stable interactions at the bottom (Fig. 2H and Supplementary Fig. 3A, B). The moderate asymmetry suggests that the bottom interfaces might be preferred for homotypic assembly, or it could also reflect the intrinsic noise from sampling multiple conformations in an all-atom MD simulation. Nevertheless, consistent with Rosetta simulations, significantly more energy was required to dissociate ASC^PYD^ from the top of the AIM2^PYD^ filament than the bottom (Fig. 2H and Supplementary Fig. 3B). Together, our in silico analyses consistently suggest that individual filaments assemble bidirectionally, AIM2^PYD^ strongly prefers to assemble homotypically, and the recognition between AIM2^PYD^ and ASC^PYD^ occurs via the type 2 interface.

### In vitro experiments corroborate in silico predictions

**Fig. 3:**
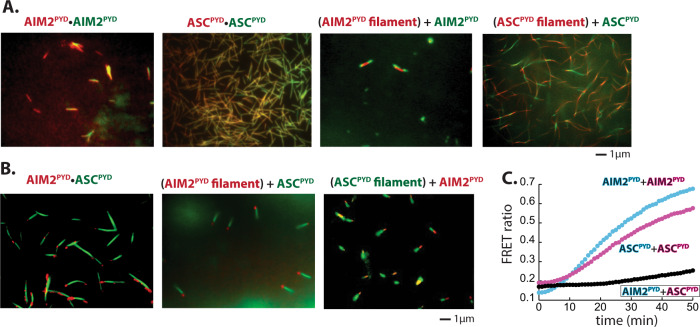
Individual filaments assemble bidirectionally and the recognition between AIM2 and ASC occurs at a specific pole. **A** Fluorescent confocal microscopy images of Alexa_488_- and Dylight_550_-labeled AIM2^PYD^, Alexa_488_- and Dylight_550_-labeled ASC^PYD^, preassembled Alexa_488_-labeled AIM2^PYD^ filament and nascent Dylight_550_-labeled AIM2^PYD^, and preassembled Alexa_488_-labeled ASC^PYD^ filament and nascent Dylight_550_-labeled ASC^PYD^. **B** Fluorescent confocal microscopy images Alexa_488_-labeled AIM2^PYD^ and Dylight_550_-labeled ASC^PYD^, preassembled Alexa_488_-labeled AIM2^PYD^ filament and nascent Dylight_550_-labeled ASC^PYD^, and preassembled Alexa_488_-labeled ASC^PYD^ filament and nascent Dylight_550_-labeled AIM2^PYD^. Images shown in **A** and **B** are representatives of at least three independent experiments. **C** The time-dependent changes in FRET ratios between donor or acceptor-labeled MBP-AIM2^PYD^ and/or MBP-ASC^PYD^ were monitored upon cleaving the MBP tag with TEVp.

To test our simulation results, we first tracked the assembly of fluor-labeled recombinant AIM2^PYD^ and ASC^PYD^ filaments via confocal fluorescence microscopy. When we mixed two populations of differentially labeled maltose-binding-protein-tagged (MBP)-AIM2^PYD^ at 1:1 ratio and triggered polymerization by cleaving MBP via Tobacco Etch Virus protease (TEVp)^19^, the two colors colocalized in the same filaments (Fig. 3A, AIM2^PYD^•AIM2^PYD^); differentially labeled ASC^PYD^ populations also colocalized in the same filaments (Fig. 3A, ASC^PYD^•ASC^PYD^). Importantly, when we preassembled the AIM2^PYD^ filament labeled with one color and added AIM2^PYD^ monomers labeled with a different color, nascent filaments extended from both axial poles of existing filaments (Fig. 3A, (AIM2^PYD^ filament) + AIM2^PYD^); ASC^PYD^ filaments also displayed random bidirectional assembly (Fig. 3A, (ASC^PYD^ filament) + ASC^PYD^). These results corroborate that homotypic filaments assemble bidirectionally. Next, we mixed differentially labeled MBP-AIM2^PYD^ and MBP-ASC^PYD^ at 1:1 ratio, and monitored their filament assembly upon triggering polymerization via TEVp. Here, each protein appeared to be oligomerized separately without colocalizing on the same filament, and two distinct filaments interacted only at one specific axial pole (Fig. 3B, AIM2^PYD^•ASC^PYD^). Such a unidirectional interaction was even more evident when we added excess nascent proteins over preformed filaments (Fig. 3B, (AIM2^PYD^ filament) + ASC^PYD^ and (ASC^PYD^ filament) + AIM2^PYD^). In addition, no significant Förster resonance energy transfer (FRET) signals were observed when we triggered the assembly of a donor-labeled AIM2^PYD^ and acceptor-labeled ASC^PYD^ (Fig. 3C. see also ref. ^19^), indicating that AIM2^PYD^ and ASC^PYD^ do not co-assemble, yet the recognition entails at least one to be oligomeric. Overall, our biochemical experiments agree with the computational predictions.

### Simulations to identify key interfaces that govern the recognition and distinction between AIM2^PYD^ and ASC^PYD^

Our observations thus far suggest that there exist distinct interactions that underpin individual assemblies and those that mediate the recognition between AIM2^PYD^ and ASC^PYD^, such as the type 2 interface for the heterotypic recognition. The side chains at the filament interfaces are poorly conserved between AIM2^PYD^ and ASC^PYD^ (Supplementary Fig. 4A), indicating that diverse side-chain interactions can support the assembly of homologous supra-structures (i.e., redundancy in assembly code). Thus, we decided to forego an alanine-scanning-like approach for identifying and validating key interfaces. Instead, we used Rosetta to suggest mutations that would selectively disrupt homotypic or heterotypic interactions without precluding filament assembly.

**Fig. 4:**
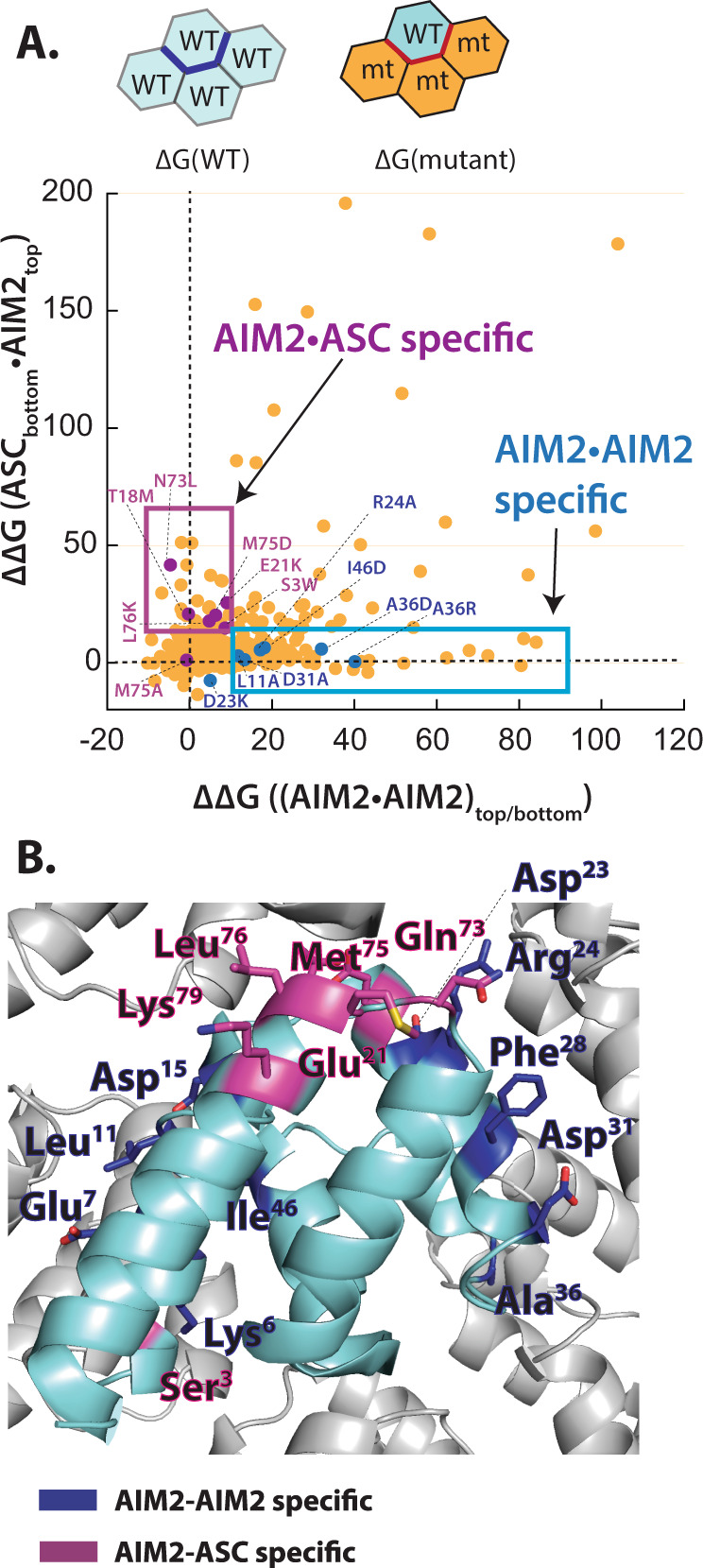
in silico mutagenesis for identifying side chains that dictate the specificity of the AIM2-ASC inflammasome assembly. **A** Top: cartoons describing in silico mutagenesis strategy. The ligand monomer was kept WT and the interface residues of pocket monomers were mutated. The ∆∆G value for each mutated residue was obtained by ∆G_(WT_ligand•WT_pocket)_–∆G_(WT_ligand•mt_pocket)_. One pocket is shown as an example, and we applied the same strategy to all six pockets described in Fig. 2 (mt: mutant). Bottom: a plot of AIM2^PYD^ mutations that would interfere with AIM2^PYD^•AIM2^PYD^ or AIM2^PYD^•ASC^PYD^ interaction. Selected mutations for follow-up biochemical and cellular studies are indicated. **B** A cartoon of the AIM2^PYD^ filament indicating the residues that Rosetta predicts to interfere with either homotypic assembly or ASC^PYD^ recognition when mutated.

Here, we in silico mutated each side chain of pocket protomers that interfaces ligand PYDs to all other a.a. using PyRosetta^42^ (e.g., Fig. 4A top). We then obtained interface energy scores (∆Gs) for WT_ligand•WT_pocket and WT_ligand•mt_pocket complexes (Fig. 4A top. WT: wild type, mt: mutant); subtracting the ∆G of each WT•mt pair from that of the WT•WT pair then provided the effect of a given mutation (∆∆G). We then plotted ∆∆Gs for AIM2^PYD^•AIM2^PYD^ (both top and bottom docking; Fig. 4A) vs. ∆∆Gs for AIM2•ASC complexes (ASC^PYD^ docking on the top pockets of AIM2^PYD^; Fig. 4A). We found that the vast majority of mutations are deleterious for both AIM2^PYD^ •AIM2^PYD^ and AIM2^PYD^ •ASC^PYD^ interactions (the upper right quadrant in Fig. 4A), suggesting that the a.a. selection has already been optimized for the self-assembly and recognition. Nonetheless, we identified 88 mutations at nine unique side chains, resulting in ∆∆G_(AIM2•AIM2)_ > 10 and ∆∆G_(AIM2•ASC)_ < 10 (i.e., mutations that would selectively disrupt AIM2^PYD^•AIM2^PYD^ interactions without abolishing AIM2^PYD^•ASC^PYD^ interactions; boxed area in Fig. 4B and listed in Supplementary Fig. 4B). Interestingly, all these side chains were found on the lateral type 1 and type 3 interfaces, but none at the axial type 2 interfaces (Fig. 4B and Supplementary Fig. 4B). Next, to identify mutations that would selectively disrupt AIM2^PYD^•ASC^PYD^ interactions, we looked for those resulted in ∆∆G_(AIM2•AIM2)_ to be <10 and ∆∆G_(AIM2•ASC)_ to be >10. Here, we identified 49 mutations at ten unique AIM2^PYD^ side-chain positions, all but one located on the type 2b surface (Fig. 4B and Supplementary Fig. 4B). These results are consistent with the mechanism, in which the lateral interfaces drive the assembly of the AIM2^PYD^ filament without biasing any directions, while the recognition between AIM2^PYD^ and ASC^PYD^ occurs at the type 2 interface.

### In vitro and in cellulo experiments corroborate in silico predictions

**Fig. 5:**
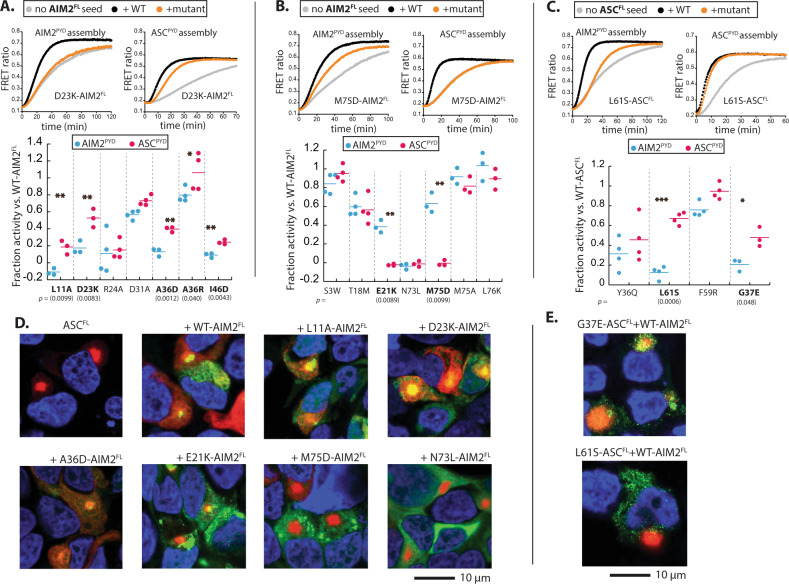
biochemical and cellular experiments support Rosetta predictions. **A** Testing AIM2^FL^ mutants predicted to interfere with homotypic assembly. **B** Testing AIM2^FL^ mutants predicted to interfere ASC^PYD^ recognition. **C** Testing ASC^FL^ mutants predicted to interfere with AIM2^PYD^ recognition. For **A**–**C**, sample plots show the time-dependent polymerization of FRET donor- and acceptor-labeled AIM2^PYD^ or ASC^PYD^ in the presence or absence of dsDNA-bound (linear plasmid ~5-kilo base-pairs (kbps)) WT or mutant AIM2^FL^. Dot plots summarizing the effect of Rosetta predicted AIM2 mutants that would selectively interfere with its own assembly. Polymerization half-times were normalized using WT AIM2^FL^-induced assembly (set to 1) and labeled PYDs alone (set to 0). *p < 0.05; **p < 0.01; ***p < 0.001. *P* values were calculated using Student’s *t* test for paired samples. **D** Confocal microscope images of HEK293T cells (co)-transfected with WT ASC^FL^-mCherry alone. WT ASC^FL^-mCherry plus WT or mutant AIM2^FL^-eGFP. **E** Confocal microscope images of HEK293T cells (co)-transfected with mutant ASC^FL^-mCherry plus WT AIM2^FL^-eGFP. The nucleus is stained with DAPI in both **D** and **E**. Images shown in **D** and **E** are representative of at least three independent experiments. .

Mutations that abolish the self-assembly of AIM2^PYD^ decreases the dsDNA-binding activity of AIM2^FL^, as oligomerization is couple to signal recognition^17^. Nevertheless, most of such AIM2^FL^ mutants still assemble into filaments on the dsDNA scaffold^17^. Also of note, AIM2 filaments can self-perpetuate its assembly by accelerating the polymerization of nascent monomers^19^. Thus, to test our simulation results, we generated Rosetta predicted mutations on AIM2^FL^ and first confirmed filament formation on dsDNA (Supplementary Fig. 5; all mutants formed filaments except N73L). We then determined whether dsDNA-bound AIM2^FL^ mutants could accelerate the polymerization of FRET donor/acceptor-labeled AIM2^PYD^ or ASC^PYD^ (Fig. 5A–C and Supplementary Fig. 6; see also ref. ^19^)

As predicted from our simulation, L11A, A36D/R, and I46D were significantly more defective in accelerating the polymerization of AIM2^PYD^ than that of ASC^PYD^ (Fig. 5A and Supplementary Fig. 6) We also tested D23K as it appeared to enhance the interaction with ASC, while disrupting AIM2–AIM2 interactions (Fig. 4A). We found that D23K-AIM2^FL^ did not enhance the interaction with ASC^PYD^, but was more defective in inducing the polymerization of AIM2^PYD^ (Fig. 5A and Supplementary Fig. 6). These results consistently suggest that the lateral surface residues of AIM2^PYD^ preferentially, but not exclusively, promote homotypic assembly. Next, again consistent with Rosetta predictions, E21K and M75D were significantly more defective in inducing the polymerization of ASC^PYD^ than that of AIM2^PYD^ (Fig. 5B and Supplementary Fig. 6), corroborating that the type 2b surface of the AIM2^PYD^ filament recruits ASC^PYD^ (N73L-AIM2^FL^ failed to induce any filament formation consistent with the lack of self-assembly (Fig. 5, and Supplementary Figs. 5 and 6)). Notably, M75A-AIM2^FL^ (null in Rosetta mutagenesis) retained the WT-like activity (Supplementary Fig. 6), supporting the idea that a simple alanine-scanning approach is inadequate due to the redundancy in assembly code.

We then used the above Rosetta-based approach to identify mutations at the type 2a surface of ASC^PYD^ that would selectively disrupt the interaction with AIM2^PYD^ (Supplementary Fig. 7A–C). Previously, we found that the ASC^PYD^ filament accelerates the assembly of AIM2^PYD^ via a positive feedback loop^19^. Thus, we generated Rosetta predicted mutations on full-length ASC (ASC^FL^) and tested their capacity for inducing the polymerization of FRET-labeled ASC^PYD^ or AIM2^PYD^. We used ASC^FL^, as the C-terminal CARD would promote the polymerization of ASC^PYD^ even if mutations were too deleterious. We found that L61S and G37E were significantly more defective in accelerating the assembly of AIM2^PYD^ than that of ASC^PYD^, corroborating that the type 2a surface of ASC^PYD^ recognizes AIM2^PYD^ (Fig. 5C and Supplementary Fig. 7D).

We next probe the interactions among AIM2^FL^ and ASC^FL^ WT and mutants in HEK293T cells. WT AIM2^FL^ (ASC^FL^) tagged with C-terminal eGFP or mCherry colocalized in the same complexes as expected (Supplementary Fig. 8). AIM2^FL^ showed filamentous complexes that often tangled up into speck-like clusters, while ASC^FL^ displayed large puncta, as previously reported^43^ (Fig. 5D left, Supplementary Fig. 8A–C). AIM2^FL^-eGFP mutants colocalized with WT AIM2^FL^-mCherry (Supplementary Fig. 8B), likely due to assembling/binding on the same (transfected) dsDNA strands as WT. Interestingly, when AIM2^FL^-eGFP and ASC^FL^-mCherry were co-transfected, ASC^FL^ filaments further expanded as if ASC^FL^ assembles from multiple AIM2^FL^ foci (Fig. 5D, (+WT AIM2^FL^)). When we co-transfected AIM2^FL^-eGFP mutants and WT ASC^FL^-mCherry, those that preferentially decreased the ability to interact with AIM2^PYD^ still resulted in expanded ASC^FL^ complexes as observed from WT (Fig. 5D, L11A, D23K, and A36D). By contrast, ASC^FL^ stayed as a single punctum when co-transfected with AIM2^FL^ mutants that failed to accelerate the polymerization of ASC^PYD^ (E21K and M75D; Fig. 5D, E21K and M75D); N73L-AIM2^FL^-eGFP, which cannot oligomerize (Supplementary Figs. 5 and 8A), also failed to interact with ASC^FL^-mCherry (Fig. 5D, N73L). We also imaged G37E- and L61S-ASC^FL^-mCherry with eGFP-tagged WT AIM2^FL^ and ASC^FL^. The ASC^FL^ mutants still showed large puncta and also colocalized with WT (Supplementary Fig. 8C). However, WT AIM2^FL^-eGFP failed to colocalize or induce the expansion of these mutants when co-transfected (Fig. 5E). Together, our in vitro and in cellulo experiments consistently support our in silico predictions, in which unique lateral and axial interfaces within homologous filaments dictate their recognition and distinction.

## Discussion

Inflammasomes transduce signals by assembling supramolecular structures^14,17–20,22,23^. It is well accepted that architectural complementarity is important for the recognition between primary signaling components^21–23,28,30,34^. Indeed, our AIM2^PYD^ filament structure further cements this concept. Nevertheless, these observations further highlight a long-standing question as to how such homologous filaments distinguish and recognize one another. Here, we set forth a broadly applicable research platform for answering these questions and propose design principles that define the signaling mechanisms of inflammasomes.

### Strategies for homotypic filament assembly

**Fig. 6:**
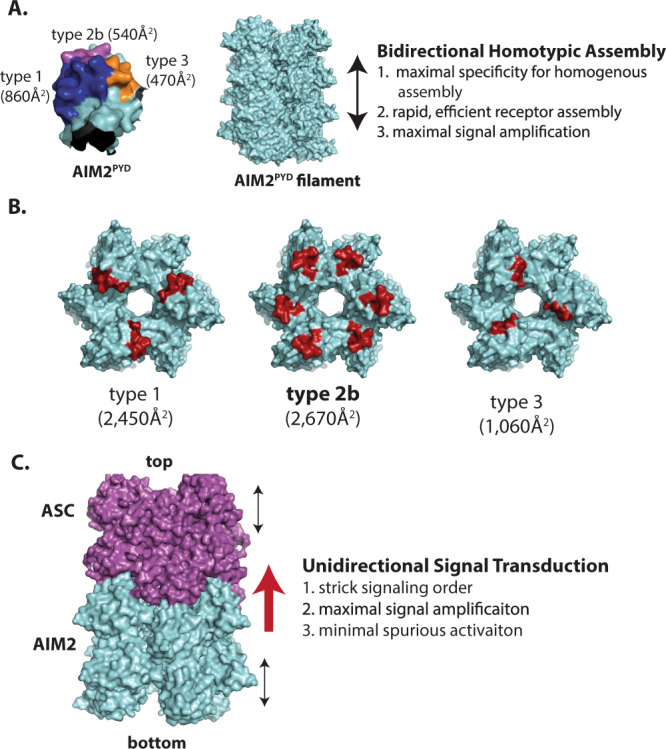
Strategies for signal transduction by the AIM2-ASC inflammasome. **A** Left: a surface representation of AIM2^PYD^ monomer. The buried surface area in the filament for each interface type is indicated. Right: a scheme describing the advantages of bidirectional homotypic assembly. **B** The top surface view of the AIM2^PYD^ filament. Each solvent accessible interface is colored in red with the calculated surface area. **C** A scheme describing the advantages of unidirectional signal transduction by the AIM2-ASC inflammasome.

Our experiments consistently show that the assembly of individual filaments occur bidirectionally, with lateral type 1 and type 3 interfaces (especially type 1) of AIM2^PYD^ favoring homotypic interactions, while still supporting the recognition of ASC^PYD^. The lateral type 1 surfaces are the largest in any PYDs (Fig. 6A left), which would be ideal for recognizing other homotypic protomers to initiate assembly without any prescribed directionalities. The lack of directionality in homotypic assembly would then allow AIM2 to maximally benefit from one-dimensional random diffusion on pathogenic dsDNA^19^, resulting in a timely response by the upstream receptor (Fig. 6A right). Interestingly, the bidirectional assembly is in contrast to other cytoskeletal and signaling filaments such as actin^44^ and B-cell lymphoma 10 (BCL10)^39^. Considering that both actin and BCL10 filaments originate from cell membranes/defined borders^39,44^, it is tempting to speculate that the bidirectional assembly of inflammasome filaments have evolved to take full advantage of no immediate boundaries in the cytosol.

### Strategies for signaling by assembly

The AIM2^PYD^ filament displays higher stability than either the ASC^PYD^ filament or AIM2^PYD^•ASC^PYD^ complex (Fig. 2), which would ensure homotypic assembly of the receptor filament especially within the dsDNA scaffold. On the other hand, the interaction between AIM2^PYD^ and ASC^PYD^ is more favorable than homotypic ASC^PYD^ interactions at a specific axial pole (Fig. 2). In addition, AIM2^PYD^ and ASC^PYD^ recognize each other only when at least one is oligomeric (Fig. 3B). Of note, electrostatic surface analyses suggest that the charge complementary is reversed at the type 1 interface for AIM2 and ASC, likely indicating that the heterologous interactions between the monomers are not favorable (i.e., the type 1a surface of AIM2^PYD^ is largely basic, whereases that of ASC^PYD^ is acidic; Supplementary Fig. 9A). Importantly, our in silico, in vitro, and in cellulo experiments consistently demonstrate that the directional interaction at the type 2 interface is most critical (Figs. 2–5). The surface area of the type 2 interface is much smaller than that of the type 1 interface in monomeric PYDs (Fig. 6A). However, because of the axial location, the type 2b surfaces become as accessible as the type 1 surfaces once AIM2^PYD^ assembles into a filament (Fig. 6B). Moreover, electrostatic surface analyses suggest that the bottom of the AIM2^PYD^ filament is unfavorable for interacting with the ASC^PYD^ filament due to highly positively charged surfaces (Supplementary Fig. 9B). We propose that such conditional scaffolding by the upstream filament not only ensures proper signal transduction orders, but also maximizes signal amplification (Fig. 6C). For instance, inflammasomes assemble in a digital fashion and entail cell death^14,19^. Thus, it must be imperative that ASC does not polymerize unless upstream receptors are fully activated by correct signals. Thus, the sequential/conditional assembly of the AIM2-ASC inflammasome would minimize its spurious activity in the absence of bona fide danger signals. Of note, not only the assembly, but also the signaling activity of AIM2 depends on dsDNA length, which regulates the probability of assembling the intact axial base of its filament^19^. Our results here further explain the dsDNA length-dependent mechanism, as the intact filament base would conditionally maximize the presence of the type 2b surfaces to recruit ASC (Fig. 6B). Subsequently, such a stringent recognition mechanism would then ensure that ASC polymerizes homogenously via its prion-like assembly mechanism^45^, resulting in maximal signal amplification (Fig. 6C). Alternatively, if AIM2 and ASC were to either co-assemble or interact without distinct energetic hierarchies, we envision that upstream and downstream oligomers would either alternate or even cap their respective assemblies, resulting in signal attenuation.

### Future directions

Our successful implementation of Rosetta to decode the specificity of the AIM2-ASC inflammasome suggests that our approach can be broadly applied to other homologous signaling filaments. However, we noted that Rosetta was correct at ~50% in predicting energetically important mutations (Fig. 5), indicating that there is room for improvement. Nonetheless, given that precisely pinpointing the role of individual residues is intrinsically challenging, we find the Rosetta suite to be an excellent tool for decoding the specificity of the filamentous assemblies.

It was recently postulated that the directionality of the NLRP6^PYD^-ASC^PYD^ interaction would be the same as what we found here for AIM2^PYD^-ASC^PYD^ (ref. ^20^). Thus, it is tempting to speculate that recruiting ASC via its type 2a surface by the type 2b surface of upstream receptors is the universal strategy in inflammasome signaling. Other than NLRP6 and AIM2, there are at least 14 other upstream receptors that signals through ASC^1,14^. Future investigations will reveal to what extent the mechanisms we found for AIM2 apply in other receptors and how well Rosetta fares in answering these questions.

## Methods

### Protein expression and purification

Human AIM2^FL^ (residues 1–343), AIM2^PYD^ (residues 1–94) S94C for fluorophore labeling, ASC^PYD^ (residues 1–92) were cloned into the pET28b vector (Novagen) with an N-terminal MBP tag and TEVp recognition site. For cryo-EM, we and found a construct including ~20 a.a. in the unstructured linker region (residues 1–117) resulted in well-separated filaments (denoted as AIM2^IRND^; Fig. 1A). ASC^FL^ was cloned with the MBP tag at both N- and C-termini with the TEVp recognition site flanking MBP and ASC^FL^. All proteins were expressed in *Escherichia coli* BL21 Rosetta2^DE3^ and purified using affinity (MBP/amylose), cation exchange, and followed by size-exclusion chromatography. Proteins were then concentrated and stored at −80 °C, see also refs. ^17,19^ (all primers generated for this study are listed in Supplementary Table 2).

### Cryo-EM sample preparation

A total of 5 µl sample of 2.75 µM AIM2^IRND^ (cleaved for 30 min with 6 µM TEVp) was applied to Lacey grids, followed by automatically blotting for 1.5 s and plunge freezing, using the FEI Vitrobot Mark IV operated at 100% humidity and room temperature (Johns Hopkins University). Cryo-EM data were collected at the National Cancer Institute National cryo-EM facility (NCI NCEF, Frederick, MD) using the FEI Titan equipped with the Gatan K2 direct electron detector operating at 300 keV, using the super-resolution mode (0.66 Å/pixel). A total of 2700 micrographs were collected from one grid at a defocus range from −1.0 to −2.5 µm. Each micrograph was equally fractioned into 40 frames with a total exposure time of 12 s and a total dose of 42 electrons/Å^2^. Data collection statistics are listed in Supplementary Table 1.

### Helical reconstruction and model building

The 2700 micrographs were binned by two (to 1.32 Å/px) and frames were aligned using MotionCor2 (ref. ^46^). The defocus values and astigmatism of the micrographs were determined by CTFFIND3 for the aligned full-dose micrographs^47^. A total of 976 micrographs were selected (images with good CTF determination and defocus <3 μm) for subsequent image processing. CTF was corrected by multiplying the micrographs (only first 20 frames were aligned with a total dose of ~20 electrons/Å^2^) with the theoretical CTF, which both corrects the phases and improves the signal-to-noise ratio. The e2helixboxer program in EMAN2 software package^48^ was used for boxing long filaments from the full-dose images. The CTF-corrected micrographs were used for the segment extraction, with a total of 116,285 384 px-long overlapping segments (with a shift of 1.5 times of axial rise) generated. The SPIDER software package^49^ was used for subsequent processing and reconstruction. Using a featureless cylinder as an initial reference, 99,237 segments were used in IHRSR program for the final reconstruction after the helical parameters (an azimuthal rotation of 53.3° and an axial rise of 14 Å per subunit) converged. The resolution of the final reconstruction was estimated by the FSC between two independent half maps, which shows 3.2 Å at FSC = 0.143.

We used the crystal structure of AIM2^PYD^ (PDB ID: 4O7Q) as an initial template to dock into the AIM2^PYD^ cryo-EM map by rigid body fitting, and then manually edited the model in Chimera^50^ and Coot^51^. We then used the modified model as the starting template to further refine against the segmented cryo-EM map using RosettaCM^52^. The refined monomeric model of AIM2^PYD^ was then rebuilt by RosettaCM with helical symmetry and real-space refined, using Phenix^53^ to improve the stereochemistry, as well as the model-map correlation coefficient. The AIM2^PYD^ filament model was validated with MolProbity^54^. Refinement statistics are listed in Supplementary Table 1.

### Rosetta docking

The Rosetta Local Docking protocol^40,55^ was applied to Rosetta symmetry relaxed structures of the AIM2^PYD^ cryo-EM structure and ASC^PYD^ cryo-EM structure (PDB ID: 3J63). Each complex contained a pocket consisting of three PYDs, and one ligand PYD. The initial position of the ligand was already in the pocket to minimize the search space, as suggested by the local docking protocol. The docking simulation was done 5000 times for each fragment, and the results were analyzed by looking at the interface energy and RMSD from the initial position.

### Interface energy analysis and in silico mutagenesis

We used the InterfaceAnalyzer script in Rosetta at individual interfaces of the honeycomb to obtain interaction energies.

Using PyRosetta, we first in silico mutated each interface residue of all AIM2^PYD^/ASC^PYD^ protomers in the honeycomb into all other possible a.a.^42^. We then removed those that resulted in energy scores >2 standard deviations from the mean (e.g., prolines that would distort backbone geometry and cause potential folding issues). We then applied remaining mutants (942 possibilities) to the pocket protomers of all six subsections, leaving the ligand protomers as WT. We then used the dG_separate subroutine in Rosetta InterfaceAnalyzer to obtain ∆Gs for (WT_ligand•WT_pocket) and (WT_ligand•mutant_pocket) interfaces; subtracting the ∆G of each mutant from that of WT then provides the changes in interface energy caused by the mutation (∆∆G). Each mutant was tested at least three times and the average values were used for analyses.

### Molecular dynamics

The coordinates for each protein–ligand complex were obtained from Rosetta docking experiments such that the interface energy score and interface RMSD were minimized. All MD simulations were performed using GROMACS 5.1.3 (gromacs.org) with an all-atom CHARMM36 (ref. ^56^) force field. Simulation scripts were created using CHARMM-GUI^57,58^. Initial coordinates were energy minimized using the steepest descent algorithm and subsequently equilibrated for 4.75 ns, starting in the NVT ensemble and transitioning to the NPT ensemble. Neutralizing ions were added with ~200 Cl and ~200 K to a box of 13 nm × 13 nm × 13 nm.

Following initial energy minimization and equilibration, a second step of equilibration was performed in the NPT ensemble with a 2 fs timestep for 50 ns. A Nose-Hoover^59^ thermostat was used to maintain a reference temperature of 300 K with a 1 ps coupling time constant. The protein and solvent were coupled to separate temperature baths. A Parrinello-Rahman^6^ isotropic barostat with a 5 ps coupling time constant was used to maintain a pressure of 1 bar. Particle Mesh Ewald (PME) with a 1.2 nm cutoff radius, a 0.12 nm Fourier spacing, and cubic interpolation of 4 were used for electrostatics^60^. Van der Waals interactions had a 1.2 nm cutoff radius. A LINCS algorithm was used for bond constraints and XYZ periodic boundary conditions were enforced^61^.

Following the second step of equilibration, well-tempered Metadynamics (MetaD) simulations were performed using GROMACS 5.1.3 patched with PLUMED2 (ref. ^62^), using a CHARMM36 force field. The collective variable (CV) was the distance between the center-of-mass of the pocket and the center-of-mass of the ligand (residue 60). The backbone RMSD stayed mostly constant during the course of simulation (Supplementary Fig. 3A). Gaussians of energy were deposited along the trajectory in this CV space. Gaussians had an initial hill height of 1 kJ/mol and a width of 0.05 nm. Gaussians were deposited every 400 fs. A bias factor of 4 was used to adjust the hill heights according to the well-tempered MetaD scheme. Gaussians were saved to a grid with a bin spacing 0.01 nm. Simulations were considered complete when the ligand completely dissociated from the pocket, i.e., the CV distance exceeded 5 nm. Positional restraints were placed on every alpha-carbon in the pocket to prevent dissociation of the pocket protomers during the entirety of the MetaD simulations. The sum of all deposited Gaussians was computed to represent the dissociation free energy.

### Polymerization assays

A total of 100 nM of AIM2^FL^ was cleaved by 6 µM TEVp for 20 min in a 384-well plate. After cleavage, 150 nM of linearized plasmid dsDNA (~5-kbps, binding site normalized) was added and allowed to bind for 30 min. To start the assay, 2 µM FRET mix of MBP-tagged AIM2^PYD^ or ASC^PYD^ was added to the same well containing TEVp. Each experiment consisted of a control well with no AIM2^FL^, one with AIM2^FL^ WT, and multiple AIM2^FL^ mutants for both AIM2^PYD^ and ASC^PYD^ wells. AIM2 and ASC samples were run at the same time to ensure proper statistical analyses. Half-times for polymerization were calculated and converted to apparent kinetic rates^19,63^. The no AIM2^FL^ control and AIM2^FL^ WT control were used to normalize the kinetics rates for each mutant into an activity ratio scaling from 0 (no AIM2^FL^ present) to 1 (AIM2^FL^ WT activity). *P* values were calculated using Student’s *t* test for paired samples. The same strategy was used for ASC^FL^ WT and mutants (0.5 µM, precleaved by TEVp for 30 min) inducing the polymerization of FRET-labeled AIM2^PYD^ or ASC^PYD^.

### nsEM

AIM2^FL^ bound to dsDNA was prepared in the same manner as for the polymerization assays (100 nM protein, 150 nM dsDNA, linear plasmid ~5 kilo-bps). The samples were applied to carbon coated grids and imaged^19,64^.

### Imaging recombinant AIM2^PYD^ and ASC^PYD^ filaments

Filament assembly of Alexa_488_- or Dylight_550_-labeled MBP-AIM2^PYD^ and MBP-ASC^PYD^ (1 µM each or 3 µM of nascent proteins for Fig. 3A) was induced by removing MBP by TEVp as indicated in figure legends. For preassembly, the AIM2^PYD^ or ASC^PYD^ filament was cleaved and incubated for 30 min prior to adding nascent proteins. Images were then taken using a Zeiss Axioskop 50 with a Zeiss Axiocam HRC camera and an Axio Observer inverted microscope with LSM700 confocal module.

### Imaging AIM2^FL^-eGFP/mCherry and ASC^FL^-eGFP/mCherry in HEK293T cells

AIM2^FL^ and ASC^FL^ variants were cloned into the pCMV6 vector harboring eGFP or mCherry. To preserve native PYD–PYD interactions, the fluorescent proteins were positioned at the C-terminus of AIM2^FL^ or ASC^FL^. Plasmids were then transiently transfected into HEK293T cells using lipofectamine (0.5 µg each plasmid; Invitrogen). After 12 h, cells were fixed with 4% paraformaldehyde and mounted on glass slides using ProLong Gold Antifade Mountant with DAPI (Thermo Fisher). Cells were then imaged using the same confocal microscope as the recombinant proteins.

### Reporting summary

Further information on research design is available in the Nature Research Reporting Summary linked to this article.

## Supplementary information

Supplementary Information

Reporting Summary

## Data Availability

The cryo-EM structure has been deposited to the Protein Data Bank, PDB ID: 7K3R. The corresponding cryo-EM map was deposited in the EMDB with access code EMD-22656. The datasets generated during and/or analyzed during this study are available from the corresponding author on request. Source data are provided with this paper.

## Source data

Source Data

## Code availability

All MD scripts can be found at https://github.com/nav610/a2dissocation^65^.
