# Supplementary material for: Distinct axial and lateral interactions within homologous filaments dictate the signaling specificity and order of the AIM2-ASC inflammasome
Source: Nat Commun. 2021 May 12;12:2735. doi: 10.1038/s41467-021-23045-8 (PMC8115694; doi:10.1038/s41467-021-23045-8)
Supplement: Supplementary file 1 — Supplementary Information [file 41467_2021_23045_MOESM1_ESM.pdf]

Supplementary Information

Supplementary Tables 1-2
Supplementary Figures 1-9

Supplementary Table 1. Cryo-EM Data collection, processing and model refinement statistics of the AIM2^{PYD} filament

**Data collection and processing**	
Microscope	Titan Krios
Camera	K2
Voltage (kV)	300
Electron exposure (e^{-} Å^{-2})	42
Pixel size (Å)	0.66
Defocus range (µm)	-1.0~ -2.5
Helical rise (Å)	14.0
Helical twist (°)	54.4
Map resolution (Å)	3.2
Map B-factor	-100
**Refinement and Model validation**	
Bond lengths rmsd (Å)	0.007
Bond angles rmsd (°)	0.733
Clash score	6.35
Poor rotamers (%)	0
Ramachandran Favored (%)	97.85
Ramachandran Outlier (%)	0
MolProbity score	1.38

Supplementary Table 2

List of primers used in this study

For the IRND construct (QuikChange)

- Fwd (AIM2 IRND TAAx2 fwd): GCC ATC AGA AAT GAT TAA TAA GTC GCA AAG CAA CGT G
- Rev (AIM2 IRND TAAx2 rev): CAC GTT GCT TTG CGA CTT ATT AAT CAT TTC TGA TGG C

AIM2 Mutant primers (using the round-the-horn PCR method, unless noted otherwise):

S3W:

- Fwd: TGG AAA TAC AAG GAG ATA CTC TTG
- Rev: CTC CAT ACC ACC GGT AC

T18M:

- Fwd: ATG GAT GAG GAA CTG GAT AGG
- Rev: GAT GTT ATC CAG GCC TGT TAG C

E21K:

- Fwd: AAA CTG GAT AGG TTT AAG TTC TTT C
- Rev: CTC ATC AGT GAT GTT ATC CAG

D23K:

- Fwd: AAA AGG TTT AAG TTC TTT CTT TCA GAC GAG
- Rev: CAG TTC CTC ATC AGT GAT GTT ATC C

R24A:

- Fwd: GCA TTT AAG TTC TTT CTT TCA GAC GAG TTT AAT ATT GC
- Rev: ATC CAG TTC CTC ATC AGT GAT GTT ATC C

D31A (QuikChange):

- Fwd: GGT TTA AGT TCT TTC TTT CAG CCG AGT TTA ATA TTG CCA CAG G
- Rev: CCT GTG GCA ATA TTA AAC TCG GCT GAA AGA AAG AAC TTA AAC C

A36R:

- Fwd: AGA ACA GGC AAA CTA CAT ACT GCA AAC
- Rev: AAT ATT AAA CTC GTC TGA AAG AAA GAA CTT AAA CC

A36D:

- Fwd: GAC ACA GGC AAA CTA CAT ACT GCA AAC
- Rev: Same as A36R rev primer

N73L:

- Fwd: CTC TAT ATG CTT TTG GCA AAA CGT C
- Rev: CAA CTT CTG AAA AAT ACG AAT GGT C

N73V:

- Fwd: GTC TAT ATG CTT TTG GCA AAA CGT C
- Rev: Same as N73L Rev primer

M75D:

- Fwd: GAC CTT TTG GCA AAA CGT C

- Rev: ATA ATT CAA CTT CTG AAA AAT ACG

M75A (Quikchange):

- Fwd: GTA TTT TTC AGA AGT TGA ATT ATG CGC TTT TGG CAA AAC GTC
- Rev: GAC GTT TTG CCA AAA GCG CAT AAT TCA ACT TCT GAA AAA TAC

L76K:

- Fwd: AAA TTG GCA AAA CGT CTT CAG
- Rev: CAT ATA ATT CAA CTT CTG AAA AAT ACG

ASC Mutant primers:

Y36Q RTH with codon optimization:

- Fwd: GTC AAG GGC GCA TCC CGC GG
- Rev: CTT CAC GAA GCG GCA CCG ACA GCA G

G37E RTH, needs to be applied to the optimized Y36Q plasmid:

- Fwd: CGC ATC CCG CGG GGC
- Rev: TTC GTA ACC TTC ACG AAG CGG CAC

F59R RTH:

- Fwd: CGC TAC CTG GAG ACC TAC GG
- Rev: GCT GAC CAG CTT GTC GG

L61S:

- Fwd: AGC GAG ACC TAC GGC GCC
- Rev optimized: GTA GAA ACT GAC CAG CTT GTC G

Supplementary Fig. 1

A.

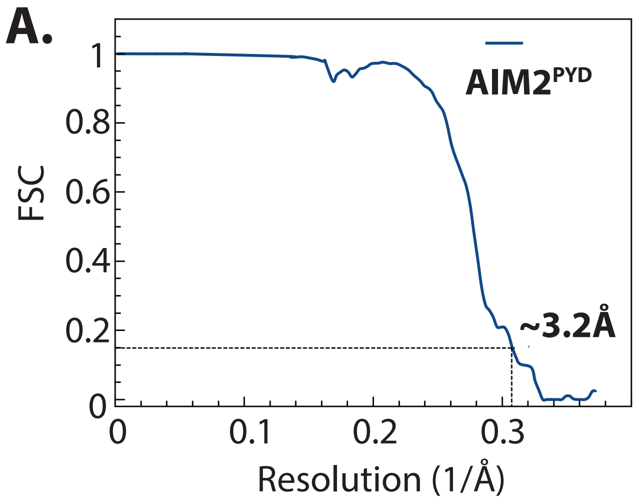

B.

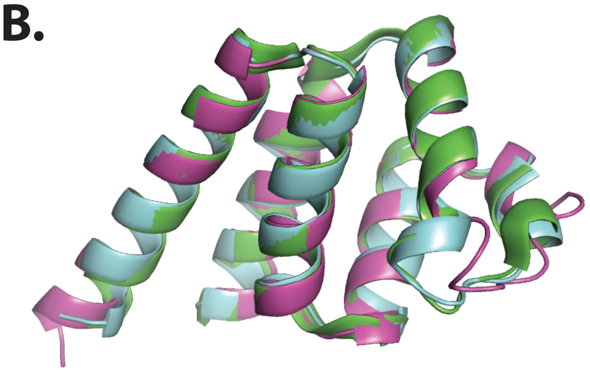

cyan: AIM2^{PYD} (filament, cryoEM)
green: AIM2^{PYD} (monomer, PDB:4o7q)
magenta: AIM2^{PYD} (monomer, PDB:3vd8)

C.

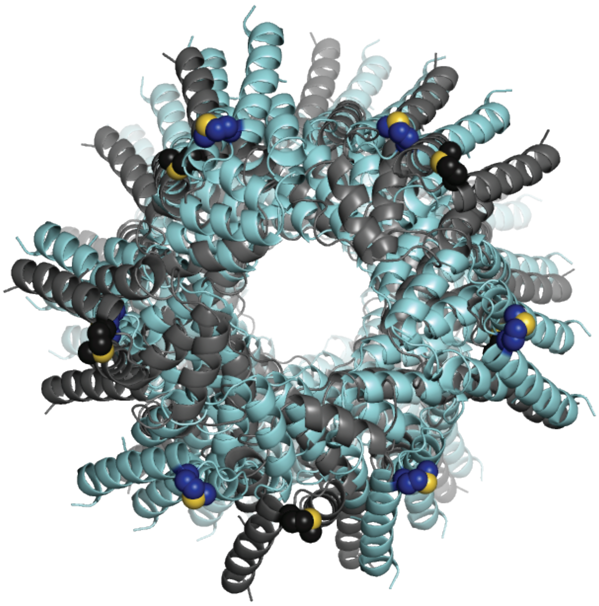

Cyan: untagged AIM2^{PYD} filament (Dark Blue: Met^{75})
Grey: GFP-AIM2^{PYD} filament (Black: Met^{75}; PDB: 6mb2)

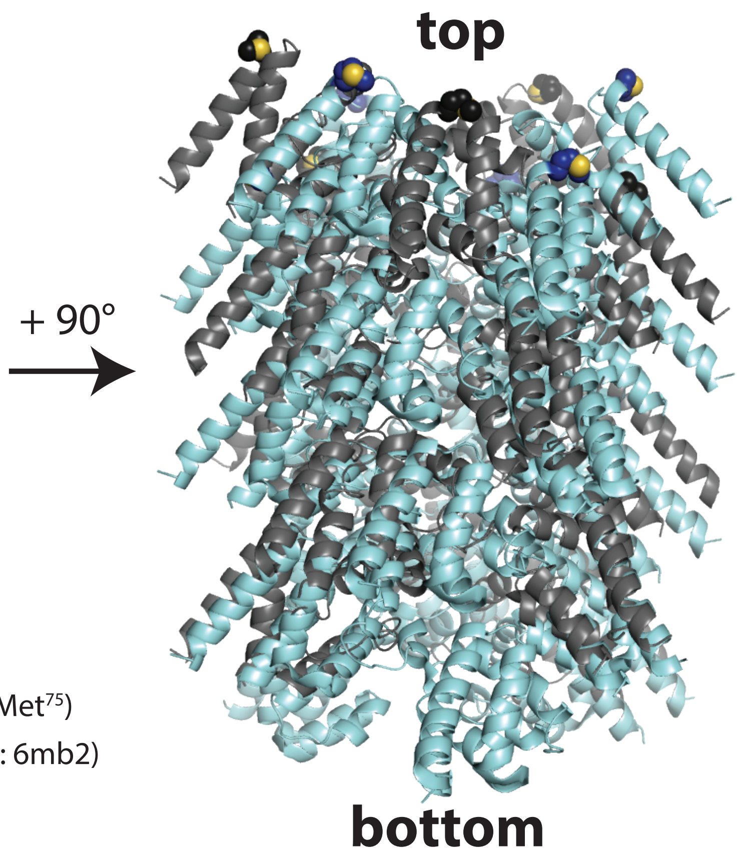

Supplementary Figure Legends

Supplementary Fig. 1

A. The FSC curve of the cryo-EM map of the $AIM2^{PYD}$ filament obtained from 99,237 segments. The dotted lines indicate the 0.143 threshold for the resolution.

B. An overlay of $AIM2^{PYD}$ subunits from the present cryo-EM structure and previously published structures.

C. An overlay of the GFP-$AIM2^{PYD}$ filament and untagged $AIM2^{PYD}$ filament.

Supplementary Fig. 2

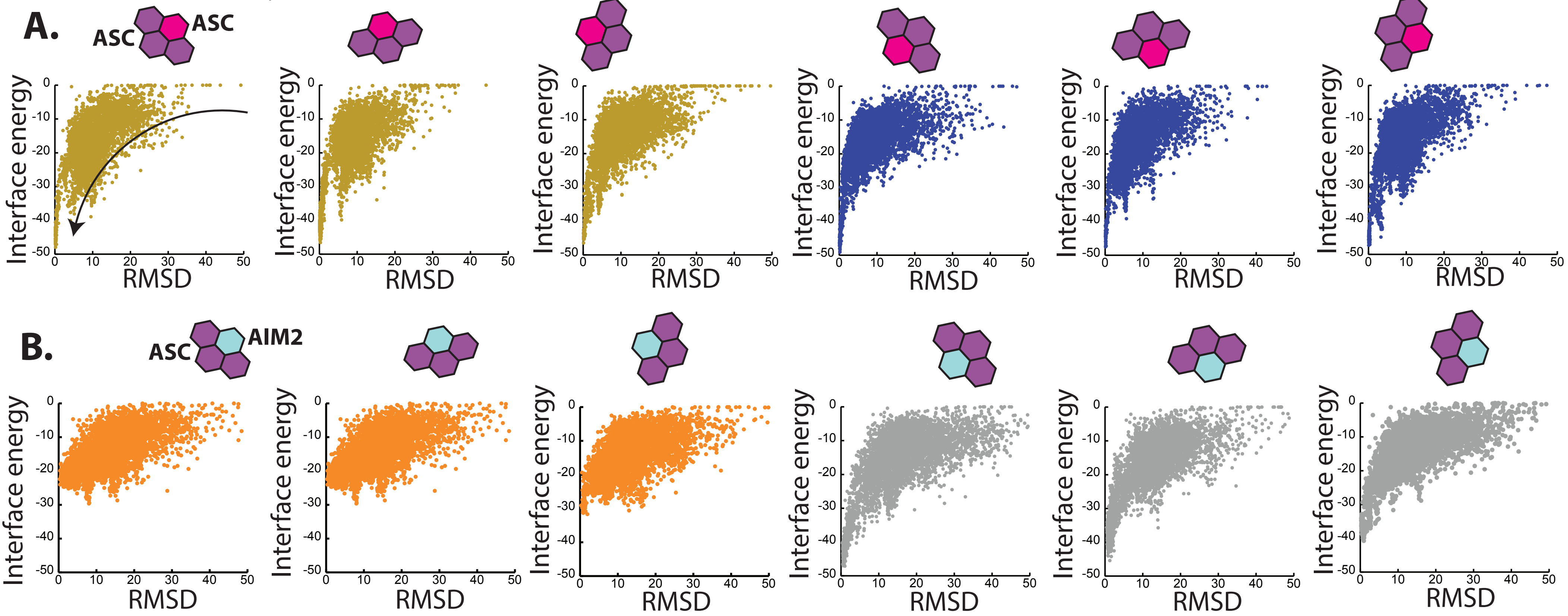

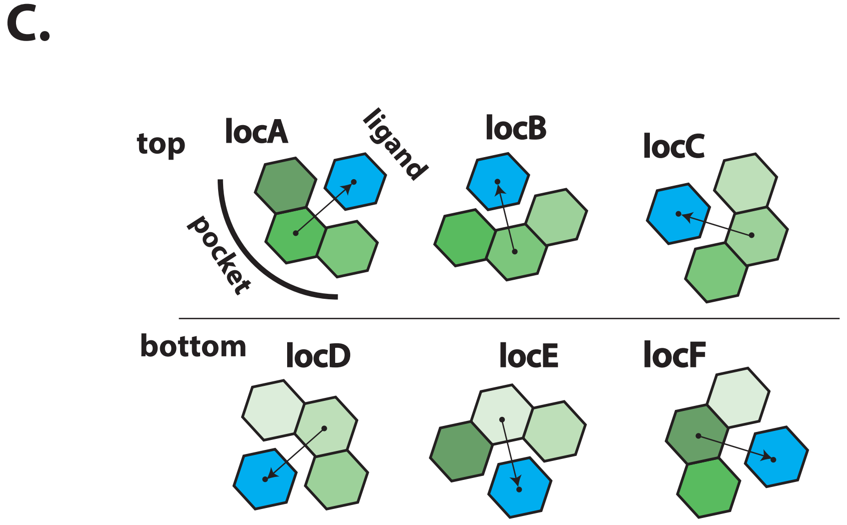

AIM2 residues: 1-94
ASC residues: 1-92

D.

**Simulation**	Equilibration Simulation Time (ns)	Metadynamics Simulation Time (ns)
AIM2-AIM2 locA	50	30
AIM2-AIM2 locB	50	50
AIM2-AIM2 locC	50	30
AIM2-AIM2 locD	50	35
AIM2-AIM2 locE	50	25
AIM2-AIM2 locF	50	35
ASC-ASC locA	50	40
ASC-ASC locB	50	50
ASC-ASC locC	50	40
ASC-ASC locD	50	50
ASC-ASC locE	50	35
ASC-ASC locF	50	50
AIM2-ASC locA	50	50
AIM2-ASC locB	50	50
AIM2-ASC locC	50	35
AIM2-ASC locD	50	40
AIM2-ASC locE	50	35
AIM2-ASC locF	50	50

Supplementary Fig. 2

A. Plots of Rosetta interface energy scores vs. RMSD for top and bottom docking results for ASC^{PYD} homotypic assembly using the published structure (PDB ID: 3J64).

B. Plots of Rosetta interface energy scores vs. RMSD for docking an $AIM2^{PYD}$ monomer on the top or bottom of ASC^{PYD} pockets.

C. A cartoon illustrating the MD simulation strategy.

D. A table summarizing equilibration and metadynamics simulation time.

Supplementary Fig. 3

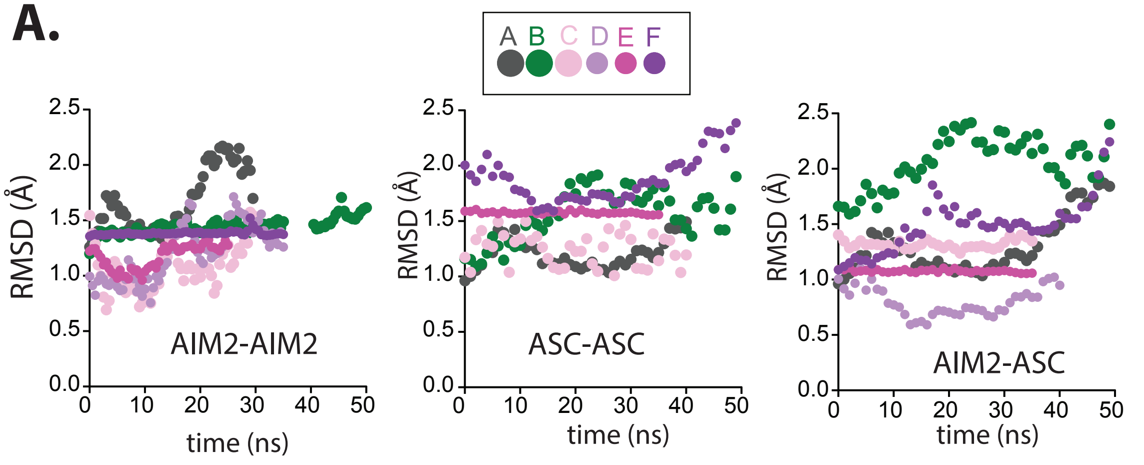

B.

	AIM2$_{lig}$-AIM2$_{pocket}$ (kcal/mol)	ASC$_{lig}$-ASC$_{pocket}$ (kcal/mol)	ASC$_{lig}$-AIM2$_{pocket}$ (kcal/mol)
Top locA	14.62	9.54	13.50
Top locB	14.89	9.11	12.48
Top locC	15.68	9.78	14.88
sum_{top} (ΔG)	*45.19*	*28.44*	*40.86*
Bottom locD	16.14	9.63	12.84
Bottom locE	15.98	9.90	11.74
Bottom locF	16.03	9.89	11.74
sum_{bottom} (ΔG)	*48.14*	*29.42*	*36.33*
$\Delta\Delta G_{top-bottom}$	-2.95	-0.98	4.53

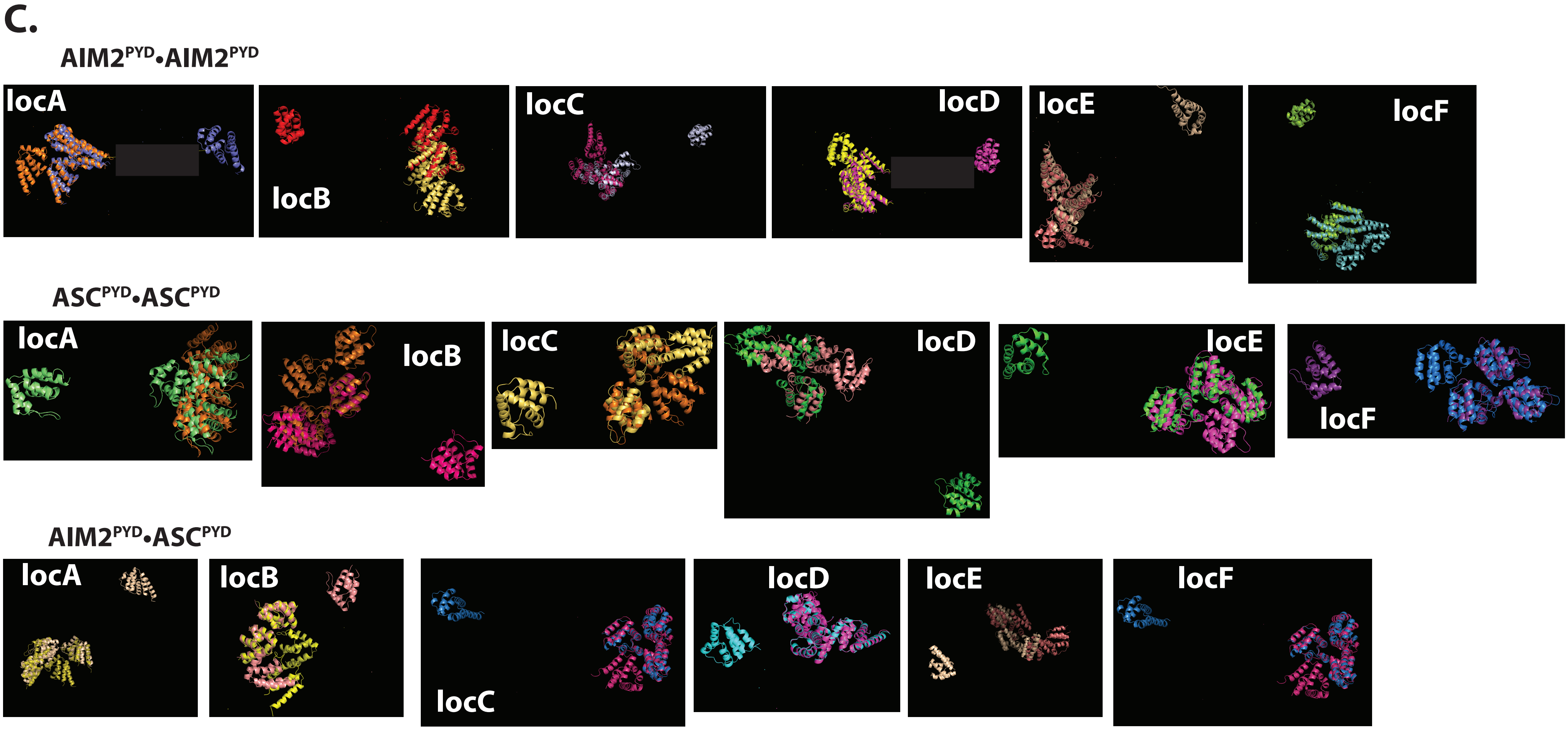

Supplementary Fig. 3

A. Plots of RMSD over simulation time (ns: nano seconds). locA-F corresponds to those shown in Supplementary Fig. 2C.

B. A table summarizing the free energy (ΔG, kcal/mol) required to dissociate a ligand PYD from each pocket.

C. Images of when each ligand was dissociated from the pocket were taken from the GROMACS trajectory.

Supplementary Fig.4

A.

```
                 10        20        30        40        50
ASC^PYD   MGR-ARDAIL-DALENLTAEELKKFKLKLLSVPLREGYGRIPRGALLSMDAL
                10        20        30          40        50
AIM2^PYD  MESKYKEILLLTGLDNITDEELDRFKFFLS-D-E---FNIATGKLHTANRIQVAT
           type 1a   type 3b    type 1b    type 2a  type 3a  type 1a

                 60        70        80        90
ASC^PYD   DLTDKLVSFYLETYGAELTANVLRDMGLQEMAGQLQAATH-Q-----
                  60        70        80        90
AIM2^PYD  LMIQNAGAVSAVMKTIRIFQKLNYMLLAKRLQEEKEKVDKQYK
             type 2a            type 2b
```

B.

AIM2 residues implicated in self-assembly	Number of mutations appeared in Fig. 4A	Interface ID
Lys6	8	Type 1a
Glu7	6	Type 1a
Leu11	12	Type 1a
Asp15	5	Type 1a
Arg24	12	Type 1b
Phe28	12	Type 1b
Asp31	11	Type 1b
Ala36	12	Type 3a
Ile46	10	Type 1b

AIM2 residues that mediate ASC recognition	Number of mutations appeared in Fig. 4A	Interface ID
Ser3	5	Type 1a
Thr18	9	Type 2b
Glu21	7	Type 2b
Asn73	5	Type 2b
Met75	11	Type 2b
Leu76	6	Type 2b
Lys79	3	Type 2b

Supplementary Fig. 4

A. An amino acid sequence alignment of ASC^{PYD} and $AIM2^{PYD}$. Side-chains that participate in each filament interface are indicated.

B. Lists of $AIM2^{PYD}$ mutations that would selectively disrupt $AIM2^{PYD}$-$AIM2^{PYD}$ (top table) or $AIM2^{PYD}$ -ASC^{PYD} (bottom table) interactions.

Supplementary Fig. 5

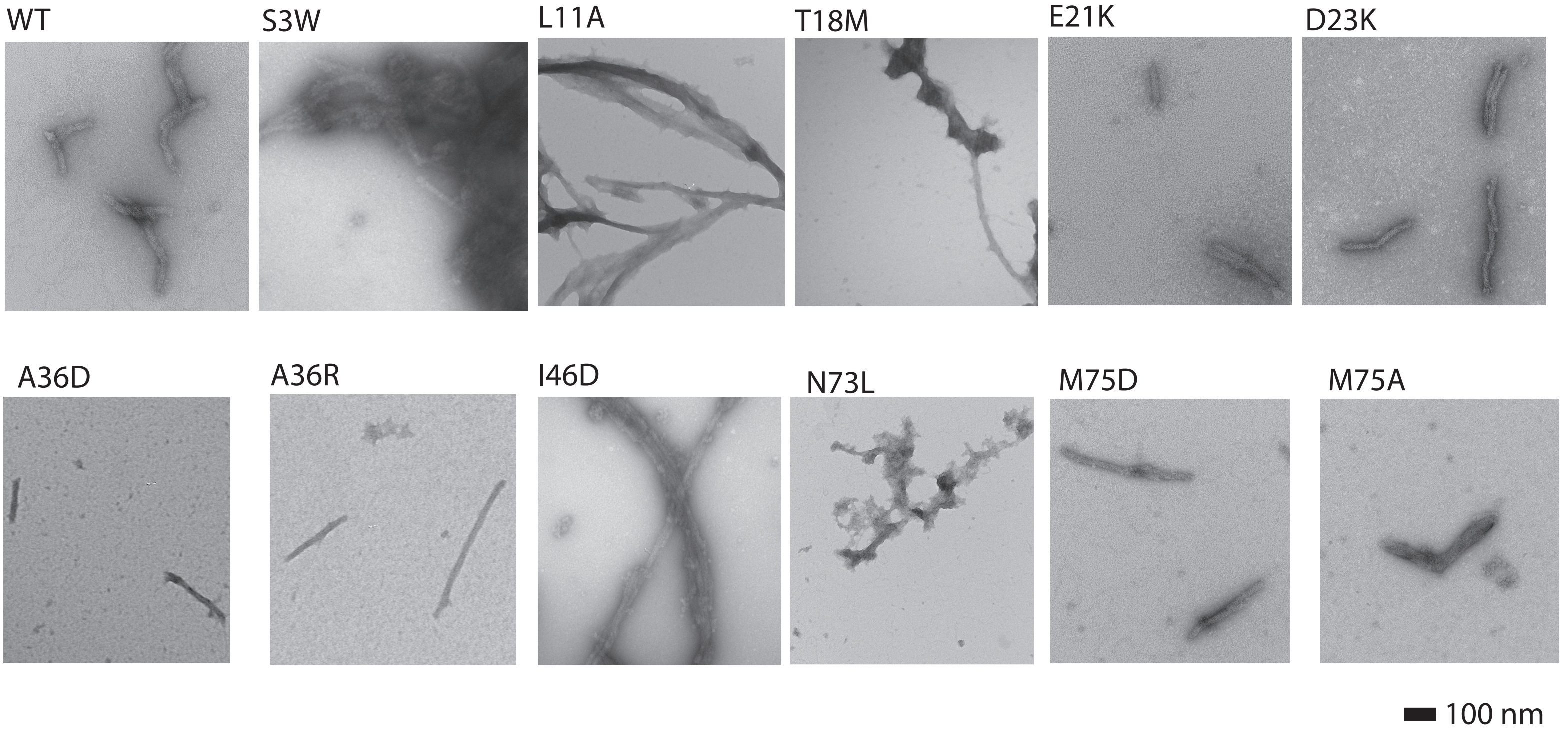

Supplementary Fig. 5

Electron micrographs of negatively stained AIM2^{FL} filaments assembled on dsDNA (linear plasmid, ~ 5-kbps).

Supplementary Fig. 6

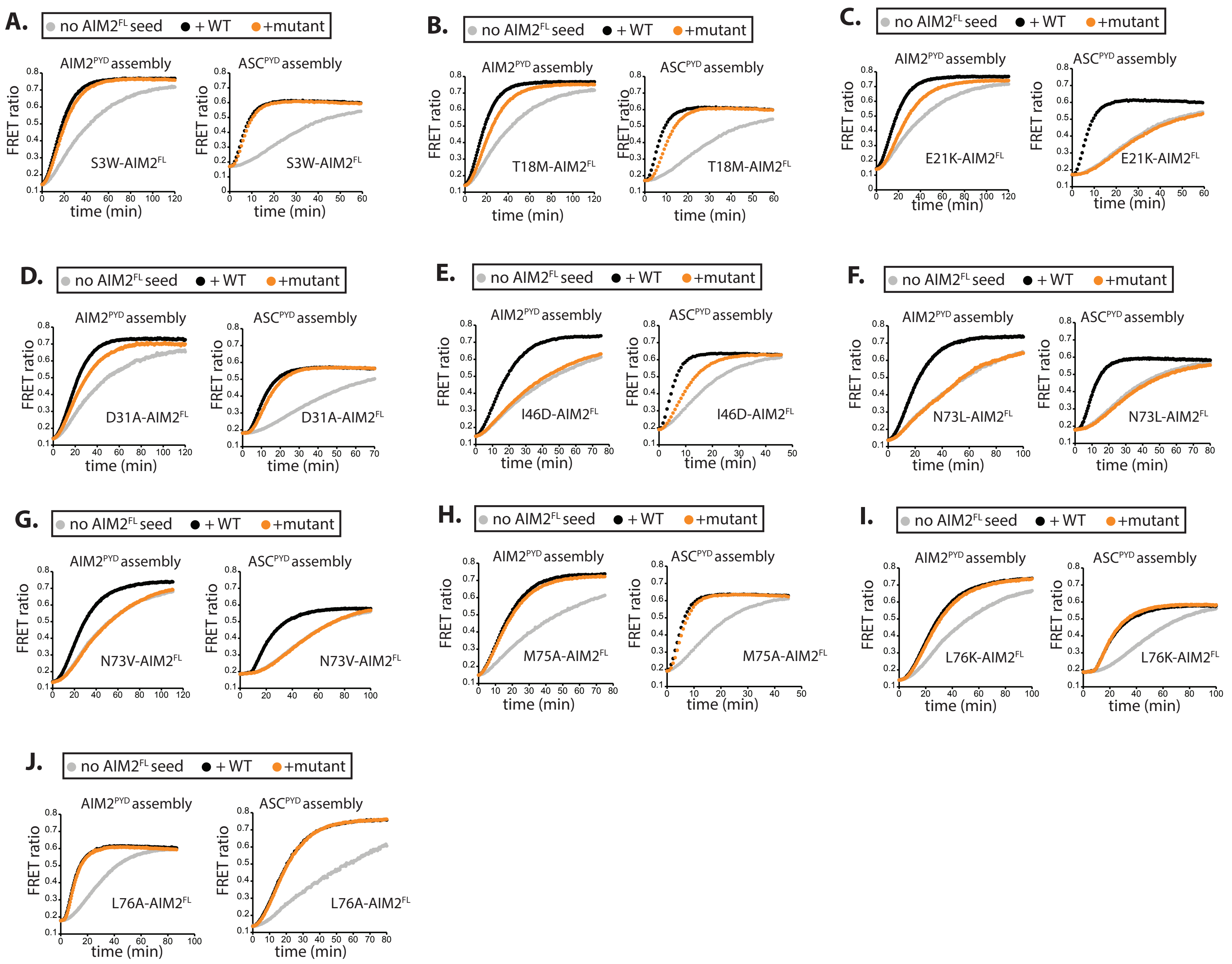

Supplementary Fig. 6

(**A-I**) Sample plots showing the time dependent polymerization of FRET donor- and acceptor-labeled $AIM2^{PYD}$ or ASC^{PYD} in the presence or absence of dsDNA-bound (linear plasmid, ~ 5-kbps) WT or mutant $AIM2^{FL}$.

Supplementary Fig. 7

A.

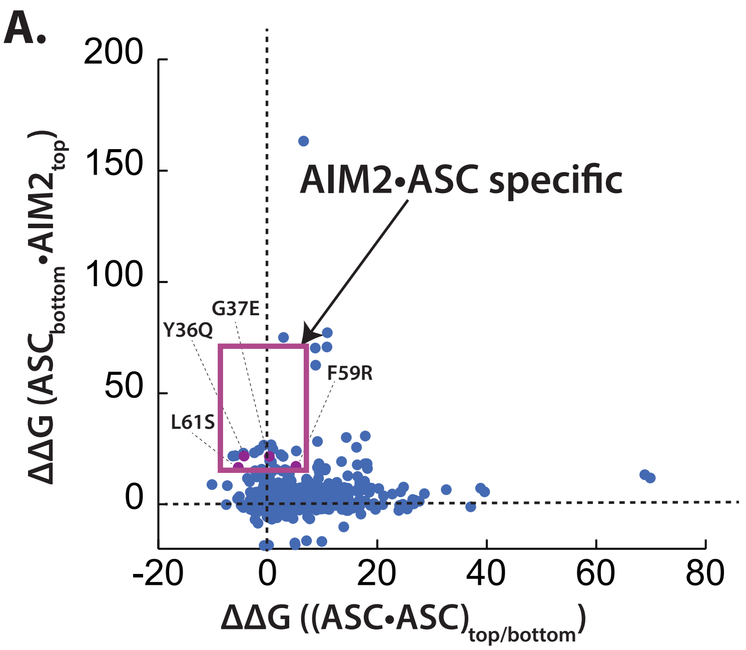

B.

ASC residues that mediate AIM2 recognition	Number of mutations appeared in A	Interface ID
Tyr36	2	Type 2a
Gly37	3	Type 2a
Pro40	3	Type 2a
Phe59	2	Type 2a
Leu61	4	Type 2a

C.

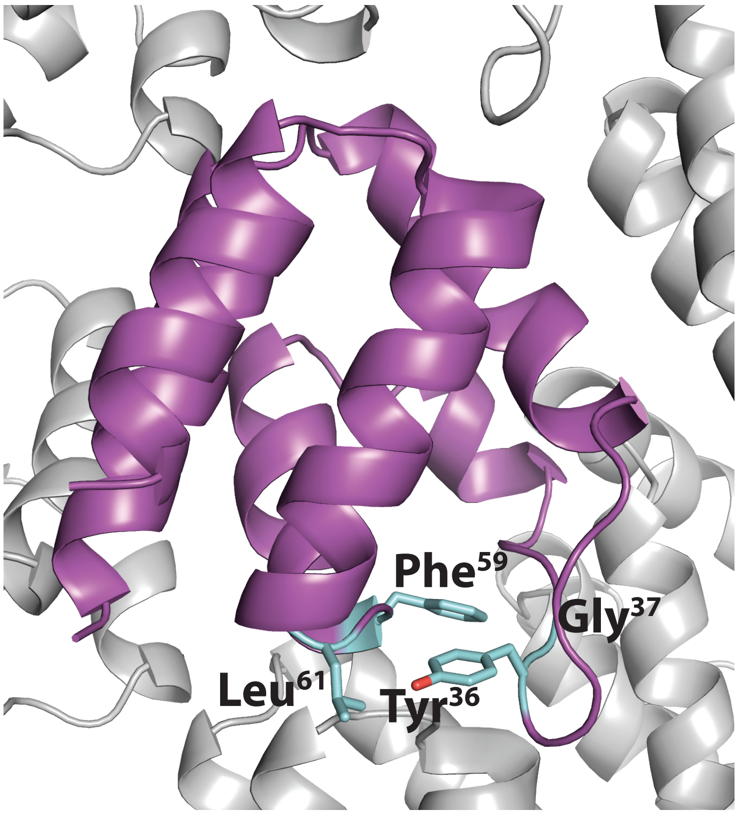

D.

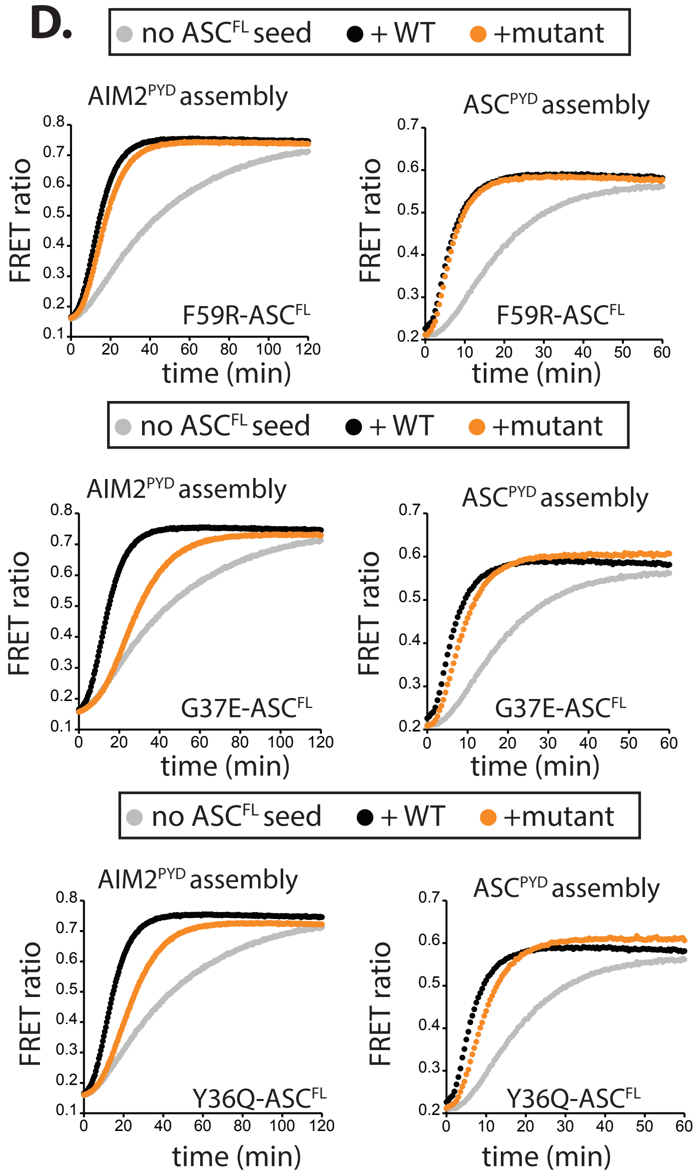

Supplementary Fig. 7

A. A plot of ASC^{PYD} mutations that would interfere with ASC^{PYD}•ASC^{PYD} or AIM2^{PYD}•ASC^{PYD} interaction. Selected mutations for follow-up biochemical and cellular studies are indicated.

B. A list of ASC^{PYD} mutations that would selectively disrupt AIM2-ASC interactions.

C. A Cartoon of the AIM2^{PYD} filament indicating the residues that Rosetta predicts to interfere with ASC^{PYD} recognition when mutated.

D. Sample Plots showing the time dependent polymerization of FRET donor- and acceptor-labeled AIM2^{PYD} or ASC^{PYD} in the presence or absence of WT or mutant ASC^{FL}.

Supplementary Fig. 8

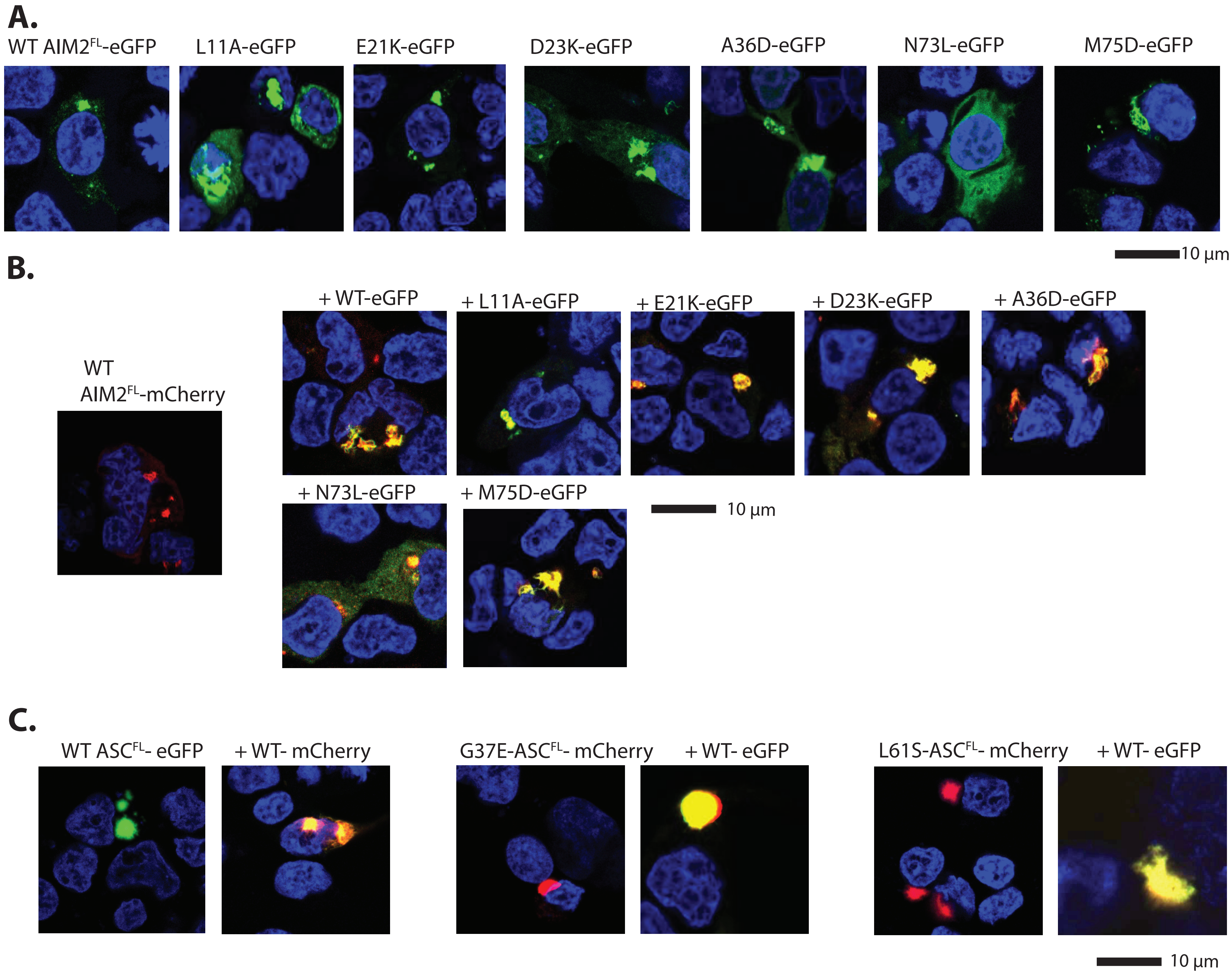

Supplementary Fig. 8

Confocal microscope images of HEK293T cells (co)-transfected with (**A**) WT or mutant AIM2^{FL}-eGFP alone; (**B**) WT AIM2^{FL}-mCherry alone, or WT AIM2^{FL}-mCherry plus various AIM2^{FL}-eGFP mutants; (**C**) From left to right: WT ASC^{FL}-eGFP alone, plus WT-ASC^{FL}-mCherry; G37E-ASC^{FL}-mCherry, plus WT-ASC^{FL}-eGFP; L61S-ASC^{FL}-mCherry alone, plus WT ASC^{FL}-eGFP. The nucleus is stained with DAPI.

Supplementary Fig. 9

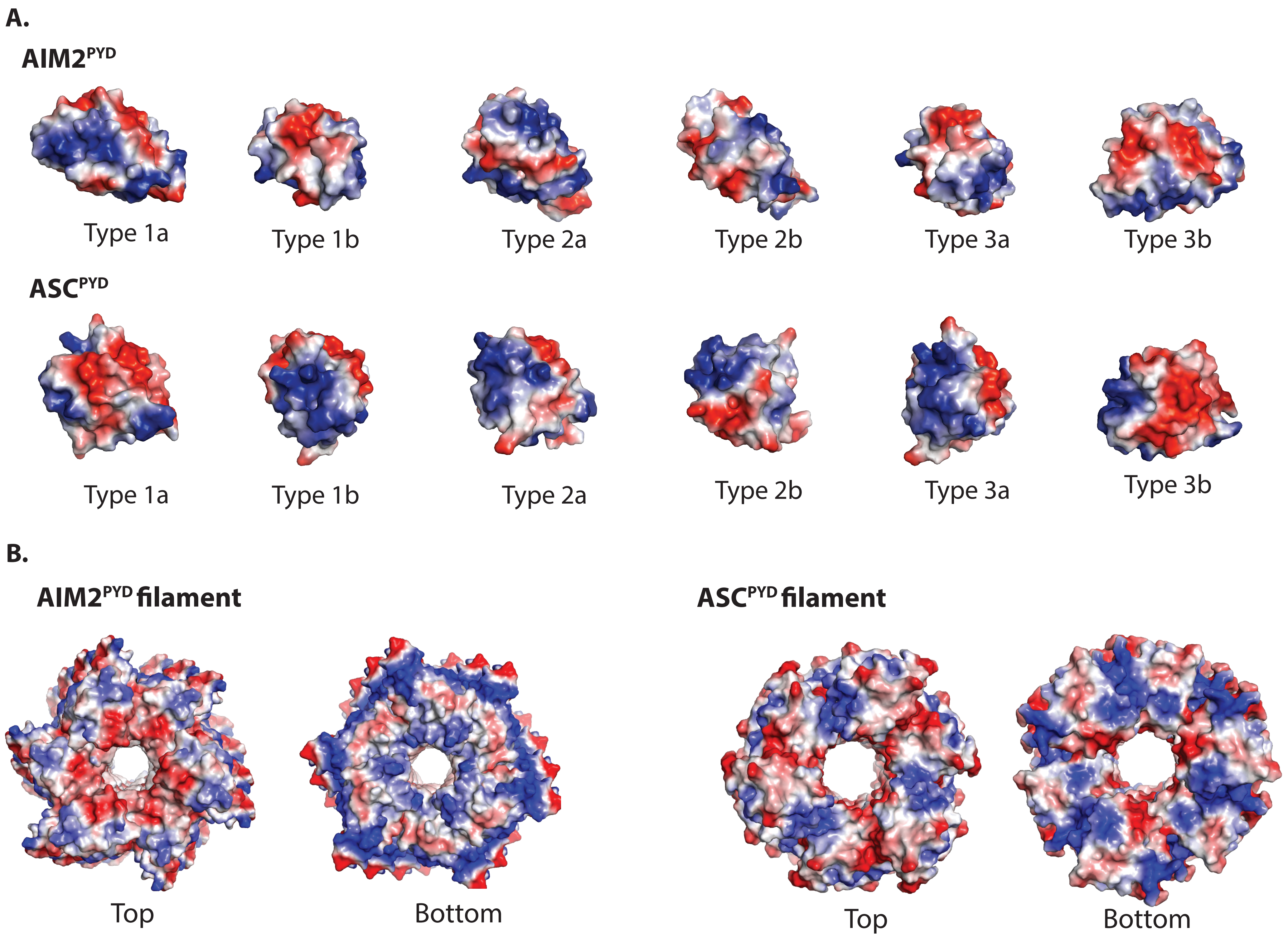

Supplementary Fig. 9

A. Electrostatic surface representations of AIM2^{PYD} (top; a monomer from the cryo-EM structure reported in this study) and ASC^{PYD} (bottom; a monomer from PDB ID: 3J63) monomers. Each surface type is shown perpendicular to the plain. Red indicates negatively charged and blue indicates positively charged residue/surface.

B. Electrostatic surface representations of AIM2^{PYD} (this study) and ASC^{PYD} (PDB ID: 3J63) filaments.
