# Supplementary material for: Distinct axial and lateral interactions within homologous filaments dictate the signaling specificity and order of the AIM2-ASC inflammasome
Source: Nat Commun. 2021 May 12;12:2735. doi: 10.1038/s41467-021-23045-8 (PMC8115694; doi:10.1038/s41467-021-23045-8)
Supplement: Supplementary file 2 — Reporting Summary [file 41467_2021_23045_MOESM2_ESM.pdf]

nature research

Corresponding author(s): Jungsan Sohn

Last updated by author(s): Apr 12, 2021

Reporting Summary

Nature Research wishes to improve the reproducibility of the work that we publish. This form provides structure for consistency and transparency in reporting. For further information on Nature Research policies, see our Editorial Policies and the Editorial Policy Checklist.

Statistics

For all statistical analyses, confirm that the following items are present in the figure legend, table legend, main text, or Methods section.

n/a	Confirmed	
☐	☒	The exact sample size (n) for each experimental group/condition, given as a discrete number and unit of measurement
☐	☒	A statement on whether measurements were taken from distinct samples or whether the same sample was measured repeatedly
☐	☒	The statistical test(s) used AND whether they are one- or two-sided<br>*Only common tests should be described solely by name; describe more complex techniques in the Methods section.*
☒	☐	A description of all covariates tested
☒	☐	A description of any assumptions or corrections, such as tests of normality and adjustment for multiple comparisons
☐	☒	A full description of the statistical parameters including central tendency (e.g. means) or other basic estimates (e.g. regression coefficient) AND variation (e.g. standard deviation) or associated estimates of uncertainty (e.g. confidence intervals)
☐	☒	For null hypothesis testing, the test statistic (e.g. F, t, r) with confidence intervals, effect sizes, degrees of freedom and P value noted<br>*Give P values as exact values whenever suitable.*
☒	☐	For Bayesian analysis, information on the choice of priors and Markov chain Monte Carlo settings
☒	☐	For hierarchical and complex designs, identification of the appropriate level for tests and full reporting of outcomes
☒	☐	Estimates of effect sizes (e.g. Cohen's d, Pearson's r), indicating how they were calculated

Our web collection on statistics for biologists contains articles on many of the points above.

Software and code

Policy information about availability of computer code

Data collection	Softwares associated with the Titan Krios microscope, the Tecan M1000 plate reader, and the Axio microscope
Data analysis	EMAN2 (2.3,1), RosettaDock (2018.33.60351) Rosetta InterfaceAnalyzer/suites (2017.26.59567) PyRosetta (2017.26.59567) GROMACS 5.1.3, CHARM36. Kaleidagraph 3.5

For manuscripts utilizing custom algorithms or software that are central to the research but not yet described in published literature, software must be made available to editors and reviewers. We strongly encourage code deposition in a community repository (e.g. GitHub). See the Nature Research guidelines for submitting code & software for further information.

Data

Policy information about availability of data

All manuscripts must include a data availability statement. This statement should provide the following information, where applicable:

- Accession codes, unique identifiers, or web links for publicly available datasets
- A list of figures that have associated raw data
- A description of any restrictions on data availability

data availability statements are appended. source data attached, PDB ID: 7k3r, EMD:22656

Field-specific reporting

Life sciences study design

All studies must disclose on these points even when the disclosure is negative.

Sample size	we performed each experiment at least twice including protein purificaiton. For quantitative analyses, we performed at least three independent experiments. images are representative of at least three independent experiments.
Data exclusions	no data was excluded
Replication	all replication efforts have been succesful
Randomization	randomization was not necessary as we test specific hypotheses using defined components
Blinding	blinding is not applicable here as we test specific hypotheses under controlled conditions

Reporting for specific materials, systems and methods

We require information from authors about some types of materials, experimental systems and methods used in many studies. Here, indicate whether each material, system or method listed is relevant to your study. If you are not sure if a list item applies to your research, read the appropriate section before selecting a response.

Materials & experimental systems

n/a	Involved in the study
☒	☐ Antibodies
☐	☒ Eukaryotic cell lines
☒	☐ Palaeontology and archaeology
☒	☐ Animals and other organisms
☒	☐ Human research participants
☒	☐ Clinical data
☒	☐ Dual use research of concern

Methods

n/a	Involved in the study
☒	☐ ChIP-seq
☒	☐ Flow cytometry
☒	☐ MRI-based neuroimaging

Eukaryotic cell lines

Policy information about cell lines

Cell line source(s)	HEK293T cells were obtained from ATCC
Authentication	HEK293T cells were authenticated using STR profile
Mycoplasma contamination	no contamination
Commonly misidentified lines (See ICLAC register)	No commonly misidentified lines
